# Designing Self‐Assembling Chimeric Peptide Nanoparticles with High Stability for Combating Piglet Bacterial Infections

**DOI:** 10.1002/advs.202105955

**Published:** 2022-03-13

**Authors:** Peng Tan, Qi Tang, Shenrui Xu, Yucheng Zhang, Huiyang Fu, Xi Ma

**Affiliations:** ^1^ State Key Laboratory of Animal Nutrition College of Animal Science and Technology China Agricultural University Beijing 100193 China

**Keywords:** nanoparticles, peptide‐based antibacterial drug, self‐assembling, structure–function relationships

## Abstract

As a novel type of antibiotic alternative, peptide‐based antibacterial drug shows potential application prospects attributable to their unique mechanism for lysing the membrane of pathogenic bacteria. However, peptide‐based antibacterial drugs suffer from a series of problems, most notably their immature stability, which seriously hinders their application. In this study, self‐assembling chimeric peptide nanoparticles (which offer excellent stability in the presence of proteases and salts) are constructed and applied to the treatment of bacterial infections. In vitro studies are used to demonstrate that peptide nanoparticles NPs1 and NPs2 offer broad‐spectrum antibacterial activity and desirable biocompatibility, and they retain their antibacterial ability in physiological salt environments. Peptide nanoparticles NPs1 and NPs2 can resist degradation under high concentrations of proteases. In vivo studies illustrate that the toxicity caused by peptide nanoparticles NPs1 and NPs2 is negligible, and these nanoparticles can alleviate systemic bacterial infections in mice and piglets. The membrane permeation mechanism and interference with the cell cycle differ from that of antibiotics and mean that the nanoparticles are at a lower risk of inducing drug resistance. Collectively, these advances may accelerate the development of peptide‐based antibacterial nanomaterials and can be applied to the construction of supramolecular nanomaterials.

## Graphical abstract

A self‐assembling peptide nanoparticle composed of a self‐assembled core unit, a hydrophobic unit, a positively charged unit, and a hydrophilic unit is reported. Peptide nanoparticles can alter bacterial membrane structure, affecting the bacterial cell cycle, ultimately leading to membrane rupture and leakage of contents. This peptide‐based nanomaterial provides a reference solution for solving the problem of antibiotic resistance.

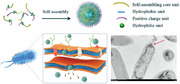

## 1 Introduction

Antibiotics have saved countless lives, so it is hailed as one of the greatest medical inventions of the 20th century. However, frequent and irregular use of antibiotics has accelerated the development of bacterial resistance.^[^ ^1^ ^]^ The SARS‐CoV‐2 pandemic has resulted in an increased usage of all antibiotics, further exacerbating the risks of bacterial resistance.^[^ ^2^ ^]^ Therefore, in recent years, the crisis of multidrug‐resistant bacteria caused by antibiotic resistance has plagued human health and animal husbandry.^[^ ^3^ ^]^ In addition, the Chinese government has issued the policy to ban the addition of feed antibiotics in feed and breeding from July 1, 2020, which has led to a significant increase in the demand for antibiotic substitutes in feed. This necessitates the development of novel antibacterial drugs or antibiotic alternatives.

As a novel type of antibiotic substitute, lipopeptides have the advantages of a broad antimicrobial spectrum, high biocompatibility, and low toxicity.^[^ ^4^ ^]^ Additionally, unlike antibiotics (that selectively interfere with specific steps of bacterial metabolism), most peptide‐based antibacterial drugs exert their antibacterial effects by physically destroying the lipid bilayers of bacteria, which makes it difficult for the bacteria to develop resistance.^[^ ^5^ ^]^ However, most peptide‐based antibacterial drugs are unstable, easily interfered with by anionic substances, and easily degraded by proteases under physiological conditions; this severely restricts the therapeutic effects of peptide‐based antibacterial drugs.^[^ ^6^ ^]^ There is an urgent need for feasible strategies to overcome the pharmacological defects of peptide‐based antibacterial drugs and improve their stability.

Supramolecular self‐assembly has provided inspiration for the development of peptide‐based antibacterial nanomaterials.^[^ ^7^ ^]^ The construction of appropriate nanostructures can produce more fascinating biological effects in peptides, which is beneficial for alleviating the pharmacokinetic defects of peptide‐based antibacterial drugs and improving their bioavailability.^[^ ^8^ ^]^ Previous reports have shown that self‐assembling nanoparticles are more effective than their free peptide counterparts, exhibiting broad‐spectrum antibacterial properties against a variety of bacteria and fungi as well as a reduced cytotoxicity.^[^ ^9^ ^]^ Additionally, self‐assembly can cause peptide‐based antibacterial drugs to achieve a stronger protease stability, which may be attributable to the protection of the cleavage site and the reduced affinity for protease.^[^ ^10^ ^]^ Most previous studies have focused on inducing the self‐assembly of natural peptides in nanomaterials, to improve performance; however, the inherent structure and function of the peptide sequence itself are often ignored, leaving very few peptide‐based antibacterial nanomaterials suitable for practical applications. In recent years, our team has focused on studying the structure–function relationships of peptide‐based antibacterial drugs, as well as the development of high‐efficiency and high‐stability antimicrobial biomaterials.^[^ ^5^, ^11^ ^]^ Therefore, it is speculated that the combination of multiple strategies, including the supramolecular self‐assembly and optimization of peptide chains, represents a promising candidate for improving the bioavailability and therapeutic effects of peptide‐based antimicrobial drugs.

**Scheme 1:**
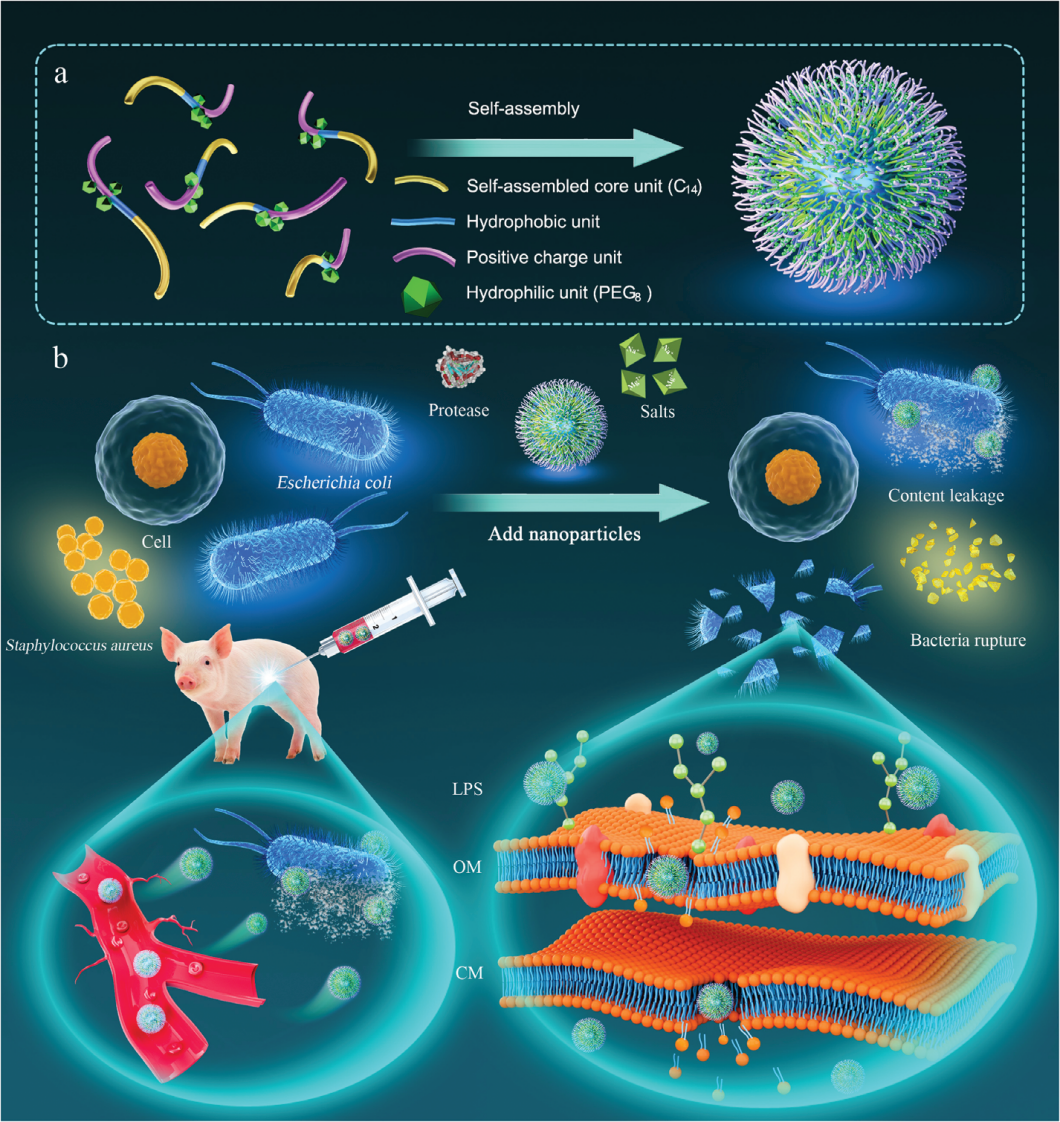
a) Schematic diagram of chimeric peptide amphiphiles self‐assembling into nanoparticles. b) Peptide nanoparticles (with excellent stability) killing bacteria through a membrane destruction mechanism. Peptide nanoparticles are injected intraperitoneally into piglets to kill bacteria. LPS: Lipopolysaccharide; OM: Outer membrane; CM: Cytoplasmic membrane.

Herein, inspired by the self‐assembly strategy,^[^ ^12^ ^]^ and based on the understanding of the structure–function relationship of peptide‐based antibacterial drugs, self‐assembling chimeric peptide nanoparticles were designed to treat bacterial infections. The peptide includes four different domains: i) A 14‐carbon alkyl chain provides hydrophobicity to the peptide and acts as a driving force for self‐assembly.^[^ ^6^, ^13^ ^]^ ii) Aromatic amino acids (phenylalanine) are used to improve the peptide's membrane interface affinity and further enhance its assembly ability.^[^ ^14^ ^]^ Prolines (Pro) are placed at each end of each phenylalanine (Phe), to limit the cleavage of the peptide chain by proteases (e.g., chymotrypsin, pepsin). iii) Cationic amino acids (lysine) impart peptide charges to ensure that the nanoparticles exert electrostatic effects on bacterial membranes.^[^ ^15^ ^]^ Similarly, Pro was used to limit Lys cleavage via proteases (e.g., trypsin). iv) A hydrophilic polyethylene glycol (PEG) domain is inserted into different positions in the peptide chain to prevent nonspecific protein adsorption, to further improve the protease stability and biocompatibility of the nanoparticle.^[^ ^16^ ^]^ Based on this design, we synthesized self‐assembled chimeric peptide nanoparticles with excellent stabilities (**Scheme** **1**). In this proof‐of‐concept study, our purpose was to evaluate the antibacterial ability, stability, and bactericidal mechanism of peptide nanoparticles in vitro, as well as to use model of bacterial infection to verify the value of our design concept.

## 2 Results and Discussion

### 2.1 Design, Synthesis, and Characterization of Self‐Assembling Peptide Nanoparticles

**Figure 1:**
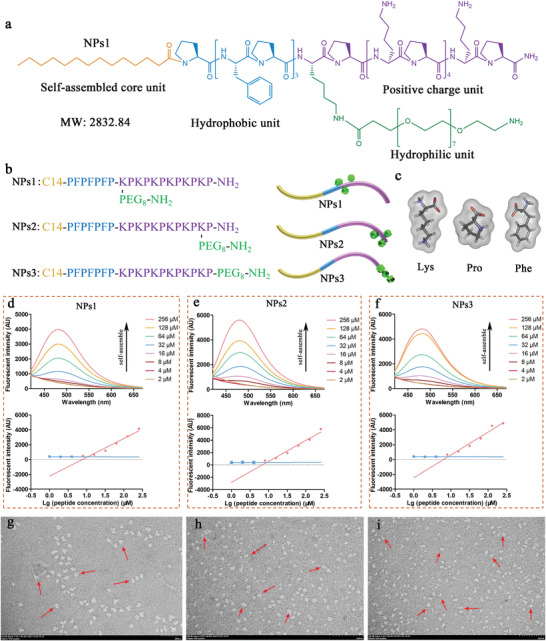
a) The NPs1 molecular structure is designed as a self‐assembling core structural unit, a hydrophobic unit, a positive charge unit, and a hydrophilic unit. b) Molecular structure and schematic diagram of peptide nanoparticles NPs1, NPs2, and NPs3. c) Structure of amino acids (Lys, Pro, Phe) constituting peptide nanoparticles. d–f) Concentration‐dependent self‐assembly and CMCs of peptide nanoparticles NPs1, NPs2, and NPs3. g–i) Transmission electron microscope (TEM) images of peptide nanoparticles NPs1, NPs2, and NPs3 at a concentration of 128 × 10^−6^ m. Scale bar: 200 nm.

Based on an understanding of the mechanism by which peptide‐based antibacterial drugs exert antibacterial activity and the principle of self‐assembly, we designed a series of self‐assembling chimeric peptide nanoparticles. It is well known that a sufficient net charge is one of the physical and chemical parameters required for peptides to undertake antimicrobial activity.^[^ ^17^ ^]^ Here, the number of positive charges in the monomer peptide was set to six, because previous studies have shown that the peptide's antimicrobial activity does not increase above this number.^[^ ^18^ ^]^ The type of positively charged amino acid was also considered. The guanidine side chain of arginine (Arg) can produce strong bidentate hydrogen bonds with the phosphate moieties of both lipid head groups; this endows the peptide with a stronger membrane disruption ability, but it also makes the loss of peptide biocompatibility and selectivity more likely.^[^ ^19^ ^]^ Furthermore, in terms of stability, Arg is more easily recognized and cleaved by trypsin than Lys.^[^ ^7^, ^20^ ^]^ Therefore, we rejected the introduction of Arg in the sequence, because it violates the design principles of high cytocompatibility and stability. As a hydrophilic carrier, PEG retains the biological functions of peptide‐based antibacterial drugs, reduces their cytotoxicity, and protects them from protease degradation in a complex physiological environment; therefore, it is often used as a cofactor in nanomaterials.^[^ ^16^, ^21^ ^]^ The charge shielding effect is one of the mechanisms by which PEG improves the cytocompatibility of nanoparticles; thus, we conjugated it to different positions of the peptide chain, to explore the effects of position on the cytocompatibility and stability of nanoparticles.^[^ ^22^ ^]^ PEG was attached to the amino group of the Lys side chain of the peptide nanoparticles NPs1 and NPs2. In NPs1, PEG was close to the hydrophobic end of the peptide chain, and in NPs2, PEG was away from the hydrophobic end of the peptide chain. In NPs3, PEG is attached to the proline at the end of the peptide chain. The terminal alkyl chains allow the peptides to form nanoparticles, and they provide the hydrophobicity required for nanoparticles to be embedded into bacterial membranes.^[^ ^23^ ^]^ It is worth noting that the type of hydrophobic amino acid also affects antimicrobial performance. Aromatic amino acids have a high membrane interface affinity, which is conducive to the penetration of peptides into the membrane.^[^ ^24^ ^]^ Additionally, Phe tends to induce peptide chain self‐assembly, and the introduction of Phe into the peptide chain skeleton helps improve the assembly abilities of supramolecular nanoassemblies.^[^ ^25^ ^]^ At the same time, all natural amino acids in the peptide chain are protected by Pro, which features a special pyrrole ring structure and can reduce the recognition and cleavage of positively charged and hydrophobic amino acids via proteases in vivo.^[^ ^25b^ ^]^ Peptide amphiphiles (NPs1, NPs2, and NPs3) were prepared via standard solid‐phase peptide synthesis techniques, according to reverse high‐performance liquid chromatography (RP‐HPLC) and matrix‐assisted laser desorption/ionization time‐of‐flight mass spectrometry (MALDI‐TOF‐MS) results. The relative molecular masses of the peptides were close to the theoretical relative molecular mass, and their purity exceeded 95%, indicating that the peptides were successfully synthesized (**Figure** **1**a–c and Figures S1–S3, Supporting Information).

The self‐assembly behavior of chimeric peptides was determined by their critical micelle concentration (CMC). Thus, the self‐assembly ability and CMC of chimeric peptides were studied using 1‐anilino‐8‐naphthalene sulfonate (ANS) fluorescence.^[^ ^26^ ^]^ As shown in Figure 1d–f, when the concentration increased, the ANS fluorescence intensities for amphiphiles NPs1, NPs2, and NPs3 increased, indicating that all chimeric peptides can form supramolecular nanostructures. Two straight lines were used to fit the ANS fluorescence intensity values of different peptide concentrations at 485 nm, and the intersection of these straight lines was used to estimate the CMC of the chimeric peptides. As shown in Figure 1d–f, the CMCs of NPs1, NPs2, and NPs3 were 10.72 × 10^−6^, 9.03 × 10^−6^, and 8.42 × 10^−6^ m, respectively, this indicates that all three peptide amphiphiles have strong self‐assembly capabilities. Subsequently, to facilitate the search for supramolecular structures, we directly observed peptide amphiphiles above the CMC (128 × 10^−6^ m) using transmission electron microscopy (TEM). The results show that peptide amphiphiles NPs1, NPs2, and NPs3 all form uniform nanoparticles with a diameter of ≈20–50 nm, and the particle sizes of peptide nanoparticles NPs1 exceed those of NPs2 and NPs3 (Figure 1g–i and Figures S4, Supporting Information). The atomic force microscopy (AFM) results show that most of the peptide nanoparticles were between 20 and 50 nm in height, essentially consistent with the TEM results (Figure S5, Supporting Information). The dynamic light scattering results show that the hydrodynamic diameter distribution of peptide nanoparticles was very wide, and several peptide nanoparticles aggregated into irregular clusters with larger particle sizes (Figure S6, Supporting Information).

Circular dichroism was used to study the secondary structures of peptide amphiphiles in different environments. In this assay, phosphate‐buffered saline (PBS) and sodium dodecyl sulfate (SDS) were used to simulate an aqueous environment and negatively charged membrane environment, respectively.^[^ ^27^ ^]^ In the PBS environment, the three peptide amphiphiles showed negative peaks at ≈205 nm, indicating that all were folded into a random coil structure. In the SDS environment, the peptide amphiphiles featured a negative peak at ≈209 nm, which also conformed to the conformational characteristics of random coils (Figure S7, Supporting Information). However, the results of circular dichroism showed that the peptide did not form a standard random coil conformation, and its maximum negative band was between 205 and 210 nm. It is possible that the peptide chain is rich in Pro, and a large amount of Pro induces the tendency of the peptide to exhibit a polyproline II helix.^[^ ^28^ ^]^ Compared with the PBS environment, the conformation of the peptide chain in the simulated membrane environment (SDS) did not vary significantly. A reasonable explanation for this is that the presence of the imino group of proline makes it difficult for the peptide to form hydrogen bonds, thereby preventing the formation of the *α*‐helix or *β*‐sheet conformation, inducing the peptide chain to form a rigid structure, and restricting the flexible movement of the peptide chains in the membrane interface.^[^ ^29^ ^]^

### 2.2 In Vitro Antimicrobial Activity, Toxicity, and Stability of Self‐Assembling Peptide Nanoparticles

**Figure 2:**
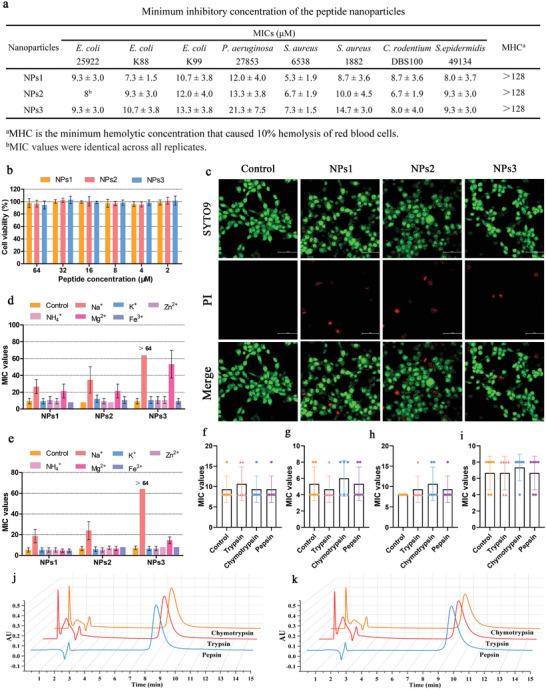
a) Minimum inhibitory concentration (MIC) of peptide nanoparticles. Each test was performed in duplicate and repeated three times. The data for each group are represented by the mean ± standard deviation (SD). b) The cytotoxicity of peptide nanoparticles to HEK293T. c) Live/dead fluorescence images of HEK293T cells treated with 64 × 10^−6^ m peptide nanoparticles. The image was taken with a super‐resolution confocal laser microscope. Scale bar: 50 µm. d,e) MIC values of peptide nanoparticles against d) *E. coli* ATCC25922 and e) *S. aureus* ATCC6538 in the presence of physiological salts. The final concentrations of NaCl, KCl, NH_4_Cl, MgCl_2_, ZnCl_2_, and FeCl_3_ were 150 × 10^−3^, 4.5 × 10^−3^, 6 × 10^−6^, 1 × 10^−3^, 8 × 10^−6^, and 4 × 10^−6^ m, respectively. The data for the control group were obtained from the MIC assay. Values denote the mean ± SD, *n* = 6. f,g) The MIC of peptide nanoparticles NPs1 against f) *E. coli* ATCC25922 or g) *S. aureus* ATCC6538 after incubation for 1 h with different proteases. h,i) MIC values of peptide nanoparticles NPs2 against h) *E. coli* ATCC25922 or i) *S. aureus* ATCC6538 after incubation for 1 h with different proteases. f–i) The data for the control group were obtained from the MIC assay. Values denote the mean ± SD, *n* = 6. j,k) Peptide nanoparticles j) NPs1, and k) NPs2 were subjected to RP‐HPLC analysis after 8 h incubation with 8 mg mL^−1^ pepsin, trypsin, or chymotrypsin.

The antimicrobial activity of the self‐assembled peptide nanoparticles was determined by a minimum inhibitory concentration (MIC) assay. As shown in **Figure** **2**a, peptide amphiphiles NPs1, NPs2, and NPs3 exhibited broad‐spectrum antimicrobial activity against gram‐negative and gram‐positive bacteria such as *E. coli* and *S. aureus*. Additionally, the MIC values of peptide amphiphiles were close to or even lower than their CMCs, which was consistent with the results of previous studies.^[^ ^9^ ^]^

**Figure 3:**
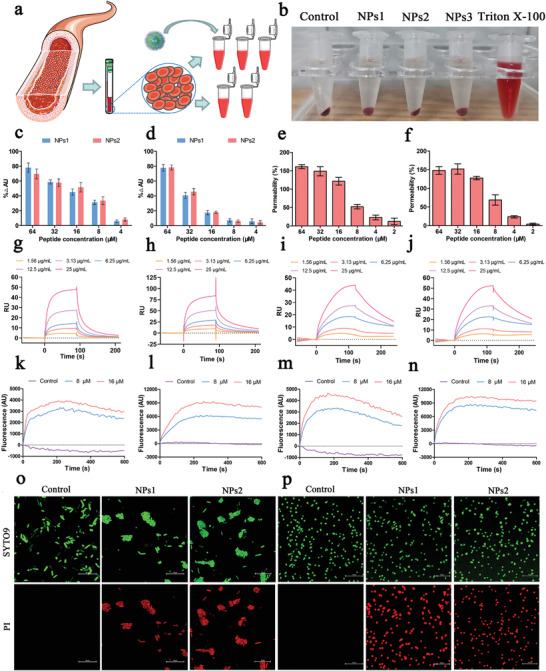
a) Schematic diagram of red blood cell collection and hemolytic activity assay. b) Photographs depicting the hemolysis of samples (Control, NPs1, NPs2, NPs3, and Triton X‐100). c) The binding affinity of peptide nanoparticles to LPS from *E. coli*. d) The binding affinity of peptide nanoparticles to LTA from *S. aureus*. e,f) The outer membrane permeability of *E. coli* ATCC25922 induced by peptide nanoparticles e) NPs1 and f) NPs2. c–f) Values denote the mean ± SD, *n* = 6. g) SPR spectroscopy of the interaction kinetics for peptide nanoparticles NPs1 and LPS. h) SPR spectroscopy results for the interaction kinetics of peptide nanoparticles NPs2 and LPS. i) SPR spectroscopy results for the interaction kinetics of peptide nanoparticles NPs1 and LTA. j) SPR spectroscopy results for the interaction kinetics of peptide nanoparticles NPs2 and LTA. k,l) The cytoplasmic membrane permeability of k) *E. coli* ATCC25922 and l) *S. aureus* ATCC6538 induced by peptide nanoparticle NPs1. m,n) The cytoplasmic membrane permeability of m) *E. coli* ATCC25922 and n) *S. aureus* ATCC6538 induced by peptide nanoparticle NPs2. o,p) Live/dead fluorescence image of o) *E. coli* ATCC25922, p) *S. aureus* ATCC6538 treated with 16 × 10^−6^ m peptide nanoparticles. Scale bar: 10 µm.

One challenge in the systemic administration of peptide‐based nanomaterials is their potential toxicity to cells and red‐blood‐cell blood compatibility. Therefore, the hemolytic activity and cytotoxicity of the self‐assembled peptide nanoparticles were evaluated. As shown in Figure 2b, no peptide nanoparticles were cytotoxic to HEK293T cells at a concentration of 64 × 10^−6^ m. Subsequently, we directly observed the cell state using a laser confocal microscope. Propidium iodide (PI) is a membrane‐permeable dye. When the cell membrane is destroyed and cell permeability increases, PI enters the cell and releases red fluorescence after embedding double‐stranded DNA.^[^ ^30^ ^]^ SYTO9 can pass through the cell membranes of living cells and bind to DNA to emit green fluorescence. As shown in Figure 2c, at a concentration of 64 × 10^−6^ m, the peptide nanoparticles NPs1, NPs2, and NPs3 showed good biocompatibility and only led to a few cell deaths. Similarly, in the hemolytic activity assay (Figure **3**a), all peptide nanoparticles showed low hemolytic activity, and the red‐blood‐cell hemolysis induced at a concentration of 128 × 10^−6^ m was negligible (Figure 3b). These results indicate that the peptide nanoparticles exhibit excellent cell compatibility in vitro. We speculate that the high cell compatibility exhibited by peptide nanoparticles is related to the “stealth effect” of PEG. Specifically, PEG attaches to nanoparticles to shield part of the charged domain; this prevents accidental physical contact with eukaryotic cells and interactions with red blood cells.^[^ ^31^ ^]^ Although PEG conjugation shields some charged domains on the nanoparticle surfaces and reduces their toxicity, the antimicrobial activity of nanoparticles is not lost. This is because the membrane structures of eukaryotic cells and bacteria are essentially different. More specifically, for bacteria, anionic lipids are exposed on the membrane surface; meanwhile, in eukaryotic cell membranes, anionic lipids are distributed inside the membrane facing the organelles.^[^ ^32^ ^]^ For this reason, cationic peptide nanoparticles can preferentially bind to bacterial membranes and achieve a better bacteria selectivity.

In a complex physiological environment, proteases and salts antagonize the antibacterial activity of peptide‐based antibacterial drugs; this has become an important obstacle to their clinical application. Specifically, monovalent, or divalent free cations (e.g., Na^+^ and Mg^2+^) competitively bind to the bacterial membrane, thereby weakening the electrostatic interaction between the peptide and bacterial membrane. Moreover, high‐valent cations (e.g., Fe^3+^) also combine with anionic groups on the surface of the bacterial membrane to increase its rigidity and thereby weaken the attack effect of the peptide thereupon.^[^ ^33^ ^]^ As shown in Figure 2d,e, Na^+^ and Mg^2+^ reduced the antibacterial activity of all peptide nanoparticles, and the remaining salts had no significant effect on antimicrobial activity. We believe that the formation of nanoparticles above the CMC of peptide amphiphiles NPs1 and NPs2, the high local density of peptide mass, and the positive charge may strengthen electrostatic interactions with the bacterial membrane, thereby weakening the effect of cations on the nanoparticles’ antibacterial activity. However, compared with NPs1 and NPs2, the salt stability of NPs3 was poor, and the antibacterial activity was almost completely lost in the presence of Na^+^. This result most likely occurred because, after NPs3 forms nanoparticles, and under the influence of PEG insertion position, PEG is located on the “shell” which completely wraps the nanoparticles; thus, the charge shielding effect of PEG affects the electrostatic interaction between the nanoparticles and bacterial membrane. Furthermore, since Na^+^ has the highest concentration of all salt, it has the greatest effect on the activity of peptide nanoparticles. In the presence of Na^+^, the antibacterial activity of peptide nanoparticles NPs1 was slightly stronger than that of NPs2, which may be due to the fact that the PEG on NPs1 is closer to the hydrophobic end, while the PEG on NPs2 is farther from the hydrophobic end, resulting in a stronger charge shielding effect.

Based on the above results, NPs3 showed poor salt stability and did not meet the selection requirements. Therefore, NPs1 and NPs2 were selected as research objects for protease stability. It is well known that cation and hydrophobicity are prerequisites for peptide‐based antibacterial drugs to exert antibacterial activity. However, cationic amino acids and hydrophobic amino acids are excellent substrates for a variety of proteases in physiological environments. For example, trypsin preferentially cleaves the C‐terminus of cationic amino acids (Arg or Lys), chymotrypsin preferentially cleaves the C‐terminus of hydrophobic amino acids (Phe, Tyr, or Leu), and pepsin preferentially cleaves the N‐terminus and C‐terminus of Phe, Leu, Trp, or Tyr.^[^ ^20^, ^34^ ^]^ Thus, in our design concept, we adopted multiple strategies to resist the cleavage of peptide nanoparticles by proteases. On the one hand, Pro was placed at both ends of the cationic amino acid (Lys) and hydrophobic amino acid (Phe), to reduce the recognition and cleavage of the proteases; on the other hand, when the concentration of peptide amphiphiles exceeded the CMC, the self‐assembly of peptide amphiphiles into nanoparticles increased the density of side chains and may reduce the accessibility of proteases.^[^ ^10^ ^]^ As shown in Figure 2f–i, after the peptide nanoparticles NPs1 and NPs2 were treated with 8 mg mL^−1^ proteases (trypsin, chymotrypsin, and pepsin) for 1 h, their antibacterial activity remained essentially unchanged, which indicates that self‐assembling peptide nanoparticles are highly stable and cannot be cleaved by proteases. RP‐HPLC was used to further determine the proteolytic stability of peptide nanoparticles. Compared with the control (treated with protease for 0 h), the chromatographic peak shape and area of the peptide nanoparticles remained intact following treatment with pepsin, trypsin, or chymotrypsin at a concentration of 4 or 8 mg mL^−1^ for 8 h, indicating that the peptide nanoparticles were very stable in the presence of protease (Figure 2j,k and Figures S8–S11, Supporting Information). In the above results, peptide nanoparticles NPs1 and NPs2 exhibit excellent stability in vitro, which lays the foundation for further in vivo research.

### 2.3 Predominant Mechanism of Self‐Assembling Peptide Nanoparticles Action

The above results show that peptide nanoparticles NPs1 and NPs2 have broad‐spectrum antibacterial activity, high cell compatibility, and excellent stability. Therefore, we regarded them as the research objects for antimicrobial mechanisms. According to the original intention of the cationic and hydrophobic design, we assumed that the action mechanism of peptide nanoparticles physically destroys the bacterial membrane, similar to most natural peptide‐based antibacterial drugs. For this reason, we chose the standard strains of gram‐negative bacteria *E. coli* ATCC25922 and gram‐positive bacteria *S. aureus* ATCC6538, to investigate the action mechanism.

The first step in the antibacterial action of peptide amphiphiles is to generate electrostatic interactions with specific components on the surface of the bacterial membrane. Lipopolysaccharide (LPS) and lipoteichoic acid (LTA) are the unique negatively charged components on the membrane surfaces of gram‐negative bacteria and gram‐positive bacteria, respectively; they are the main binding sites for peptide‐based antibacterial drugs.^[^ ^35^ ^]^ As shown in Figure 3c,d, the affinity of peptide nanoparticles NPs1 and NPs2 to LPS of *E. coli* and LTA of *S. aureus* exhibited a dose‐dependent effect, i.e., when the concentration increased, the affinity gradually increased. Moreover, peptide nanoparticles NPs1 and NPs2 exhibited a sizably enhanced binding ability to LPS and LTA at concentrations of 8 × 10^−6^ and 16 × 10^−6^ m, respectively (Figure 3c,d). This concentration was close to the CMC of peptide nanoparticles, which may relate to the increase in side‐chain cation density of the peptide via self‐assembly. To further investigate the specific binding abilities of peptide nanoparticles and LPS (or LTA), we determined the interaction of NPs1 and NPs2 with LPS (or LTA) via surface plasmon resonance spectroscopy. As illustrated in Figure 3g–j, the specific binding capacity of the peptide (as a ligand) and LPS (or LTA) (as an analyte) at concentrations of 1.56–25 µg mL^−1^ exhibited a dose‐dependent effect. The kinetic analysis results showed that the affinities KD of peptides NPs1 and NPs2 with LPS were 5.56 × 10^−5^ and 3.08 × 10^−5^, respectively, and the affinities KD with LTA were 2.65 × 10^−5^ and 1.71 × 10^−5^, respectively, indicating an evident affinity between the peptide and LPS (or LTA). Moreover, other binding parameters, including dissociation constants (*K*_d_) and binding constants (*K*_a_), are listed in Table S1 in the Supporting Information.

Gram‐negative bacteria feature a unique outer membrane that provides additional protection.^[^ ^32^ ^]^ Owing to the presence of porin, this outer membrane has a certain degree of permeability, though it is not sufficient to allow peptide‐based antibacterial drugs through. In most cases, peptide‐based antibacterial drugs can combine with negatively charged components on the outer membrane surface to remove cations, loosen the outer membrane structure, and allow larger molecules to pass through the outer membrane.^[^ ^36^ ^]^ On the other hand, the hydrophobic core of peptide‐based antibacterial drugs can be directly inserted into the outer membrane, physically destroying it. *N*‐phenyl‐1‐naphthyl amine (NPN) is a hydrophobic and sensitive fluorescent probe. Once the outer membrane structure is loose, NPN enters the cell and emits fluorescence when in contact with the hydrophobic environment.^[^ ^37^ ^]^ As shown in Figure 3e,f, peptide nanoparticles NPs1 and NPs2 penetrated the outer membrane of *E. coli* in a dose‐dependent manner. When the peptide concentration exceeded 16 × 10^−6^ m, the fluorescence release was nearly maximal, which indicated that the peptide self‐assembled into nanoparticles above the CMCs and almost destroyed the outer membrane of all *E. coli* in the suspension.

In addition to the outer membrane mentioned above, both gram‐positive and gram‐negative bacteria feature a cell wall that protects the cytoplasmic membrane.^[^ ^32^ ^]^ Therefore, DiSC_3_‐5 was used to further investigate the potential changes in the cytoplasmic membrane. This cationic dye becomes concentrated inside the cytoplasmic membrane, causing quenching of the fluorescence. When the cytoplasmic membrane potential changed, the fluorescence increased. Because the peptide nanoparticles NPs1 and NPs2 almost destroyed the outer membrane of all *E. coli* at a concentration of 16 × 10^−6^ m, we used a concentration not exceeding 16 × 10^−6^ m in this assay. As shown in Figure 3k–n, these two peptide nanoparticles induced the continuous release of fluorescence from the cytoplasmic membranes of *E. coli* and *S. aureus* within 600 s in a dose‐dependent manner. Additionally, the fluorescence increase induced by peptide nanoparticles NPs1 and NPs2 peaked in ≈200 s and then stabilized. Based on the above research results, we believe that NPs1 and NPs2 preferentially aggregate on the surface of the bacterial membrane via electrostatic interactions. When the peptide concentration exceeded the CMC, the permeability of the gram‐negative bacteria's outer membrane increased significantly; meanwhile, the degree of depolarization of the cytoplasmic membrane was increased. Similarly, for gram‐positive bacteria, peptide nanoparticles can pass through the cell wall barrier and depolarize the cytoplasmic membrane.

**Figure 4:**
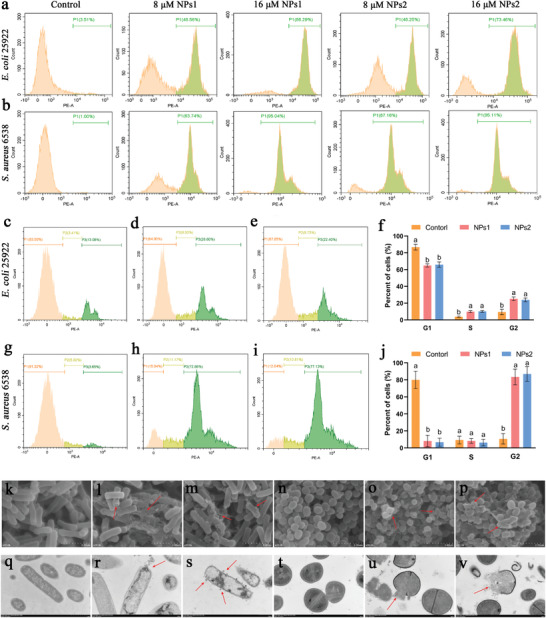
a,b) PI‐positive cells detected via flow cytometry for a) *E. coli* ATCC25922 or b) *S. aureus* ATCC6538 cells treated with peptide nanoparticles. c–e) The cell cycle of *E. coli*. bacteria: c) control, d) treated with 8 × 10^−6^ m NPs1, and e) treated with 8 × 10^−6^ m NPs2. f) Graphical representation of percentage of cell cycle in *E. coli*. g–i) The cell cycle of *S. aureus* bacteria: g) control, h) treated with 8 × 10^−6^ m NPs1, and i) treated with 8 × 10^−6^ m NPs2. j) Graphical representation of percentage of cell cycle in *S. aureus*. Values are the mean ± SD, *n* = 3. The differences between the groups were determined by one‐way ANOVA followed by Tukey's post hoc analysis. The different value as superscript indicates that there is significant difference (*p* < 0.05). k–m) SEM images of k) untreated *E. coli* ATCC25922 and *E. coli* ATCC25922 treated with 16 × 10^−6^ M peptide nanoparticles l) NPs1 and m) NPs2. n–p) SEM images of n) untreated *S. aureus* ATCC6538 and *S. aureus* ATCC6538 treated with 16 × 10^−6^ m peptide nanoparticles o) NPs1 and p) NPs2. Scale bar:2 µm. q–s) TEM images of q) untreated *E. coli* ATCC25922 and *E. coli* ATCC25922 treated with 16 × 10^−6^ M peptide nanoparticles r) NPs1 and s) NPs2. t–v) TEM images of t) untreated *S. aureus* ATCC6538 and *S. aureus* ATCC6538 treated with 16 × 10^−6^ m peptide nanoparticles u) NPs1 and v) NPs2. Scale bar: 500 nm.

To qualitatively and quantitatively verify the antimicrobial mechanism of peptide nanoparticles, laser confocal microscopy and flow cytometry were used to further analyze *E. coli* and *S. aureus*. SYTO9/PI dye was employed for live/dead staining of *E. coli* and *S. aureus*. As shown in Figure 3o,p, the green and red fluorescence of *E. coli* and *S. aureus* induced by nanoparticles NPs1 and NPs2 at a concentration of 16 × 10^−6^ m overlapped considerably, indicating that their cell membranes were severely damaged. The results of flow cytometry analysis showed that when the concentrations of peptide nanoparticles NPs1 and NPs2 approached the MIC, they caused the death of ≈50% of *E. coli* (**Figure** **4**a,b). However, doubling the concentration of peptide nanoparticles greatly increased the proportion of positive cells; this is consistent with the results of the outer membrane permeability assay, which indicates that self‐assembly after the peptide concentration exceeds the CMC will significantly enhance the destruction of gram‐negative bacterial membranes. Moreover, because *S. aureus* receives no extra protection from the outer membrane, its mortality rate exceeded that of *E. coli* after treatment with peptide nanoparticles at concentrations of 8 × 10^−6^ and 16 × 10^−6^ m. To elucidate the physiological changes induced by peptide nanoparticles in bacterial cells, we performed flow cytometry analysis of the cell cycle. As shown in Figure 4c–j, *E. coli* cells in the S and G2 phases were significantly increased after treatment with peptide nanoparticles NPs1 or NPs2, indicating that their cell cycles were arrested in the S and G2 phases. This phenomenon was more marked in *S. aureus* cells, most of which were arrested in the G2 phase after treatment with peptide nanoparticles. Therefore, the bactericidal mechanism of peptide nanoparticles is not limited to membrane lysis. Peptide nanoparticles interfere with cell metabolism, inhibit important cellular physiological activities, interfere with the normal cell cycle, and cause cell death.

Scanning electron microscopy (SEM) and TEM were used to observe the morphological changes of bacteria. The SEM results showed that the membrane surfaces of untreated *E. coli* and *S. aureus* were intact and smooth. After treatment with 16 × 10^−6^ M peptide nanoparticles, folds appeared on the surfaces of the cell membranes, and leaked contents could be observed outside the cell (Figure 4k–p). Under TEM, the bacteria in the control group exhibited a complete membrane structure and dense cytoplasm. After treatment with 16 × 10^−6^ m peptide nanoparticles, the membranes of *E. coli* and *S. aureus* were ruptured, and the cell contents were leaked (Figure 4q–v). To summarize, it was confirmed that the antibacterial mechanism of peptide nanoparticles involves multiple pathways, including penetration of bacterial membranes and disruption of the bacterial cell cycle; furthermore, the leakage of contents observed by SEM and TEM is the result of a combination of multiple factors.

### 2.4 In Vivo Biocompatibility Evaluation of Self‐Assembling Peptide Nanoparticles

**Figure 5:**
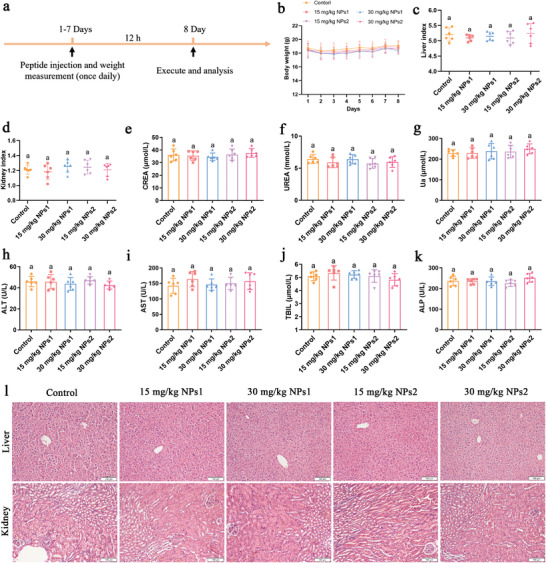
a) Schematic diagram of the in vivo mice model for peptide nanoparticle biocompatibility. b) Changes in body weight of C57BL/6 mice administration of peptide nanoparticles (0, 15, or 30 mg kg^−1^). Values are the mean ± SD, *n* = 6. c,d) Relative organ weights of c) liver and d) kidney in mice after administration of peptide nanoparticles for 7 d. e–g) Renal function index and h–k) liver function‐related indexes of mice after administration of peptide nanoparticles for 7 d. c–k) Values are the mean ± SD, *n* = 6. The differences between the groups were determined by one‐way ANOVA followed by Tukey's post hoc analysis. The same value as superscript indicates that there is no significant difference (*p* > 0.05). l) Histopathological morphology analysis of the liver and kidney in mice after administration of different concentrations of peptide nanoparticles for 7 d. Scale bar: 100 µm.

To conduct clinical trials, we injected low‐dose (15 mg kg^−1^) and high‐dose (30 mg kg^−1^) peptide nanoparticles intraperitoneally into C57BL/6 mice, and evaluated the potential adverse reactions of these peptide nanoparticles through quantitative scores, weight changes, and liver and kidney‐related indicators (**Figure** **5**a).^[^ ^38^ ^]^ After systemic administration, the mice's behavior returned to normal levels within 2 h. During the 7‐day assay period, no significant statistical difference in the body weights of the mice was observed between the groups (Figure 5b). The liver and kidney are the main sites for drug metabolism and elimination of metabolites in vivo, respectively; thus, after the end of the observation period, the relative organ weights of the mouse liver and kidney were counted. As shown in Figure 5c,d, after the assay period, no significant difference was observed in the relative organ weights between the groups of mice. Moreover, the parameters related to liver and kidney function, including creatinine (CREA), urea (UREA), uric acid (Ua), alanine aminotransferase (ALT), aspartate aminotransferase (AST), total bilirubin (TBIL), and alkaline phosphatase (ALP), were maintained at normal physiological levels, indicating that peptide nanoparticles do not induce hepatotoxicity or nephrotoxicity (Figure 5e–k). Histological examination showed that, compared with the control group, the low‐dose (15 mg kg^−1^) and high‐dose (30 mg kg^−1^) treatment groups exhibited no histological abnormalities (Figure 5l). These results show that when the intraperitoneal injection doses of peptide nanoparticles NPs1 and NPs2 were less than 30 mg kg^−1^, the side effects on mice were negligible, indicating that peptide nanoparticles NPs1 and NPs2 have good biocompatibility in vivo.

### 2.5 In Vivo Effect Evaluation of Self‐Assembling Peptide Nanoparticles

**Figure 6:**
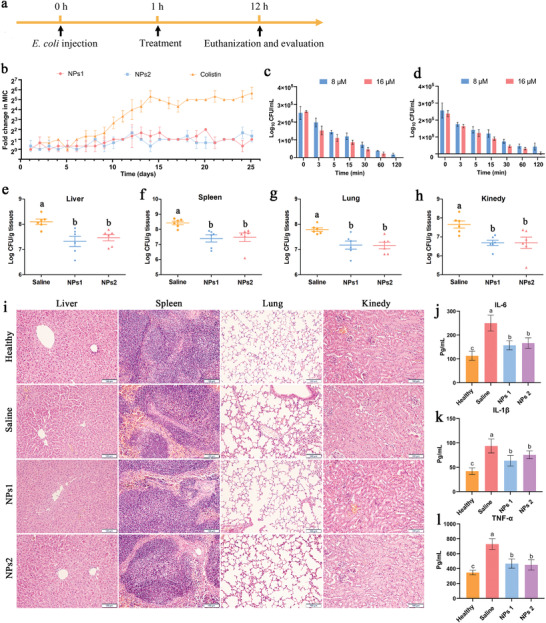
a) Schematic diagram of the experimental setup for the mice systemic infection model. b) Resistance development of *E. coli* ATCC25922 to the drugs at sub‐MIC concentrations. c,d) The killing kinetics of peptide nanoparticles c) NPs1 or d) NPs2 at 8 × 10^−6^ and 16 × 10^−6^ m concentrations against *E. coli* ATCC25922. b–d) Values denote the mean ± SD, *n* = 3. e–h) Bacterial loads of liver, spleen, lung, and kidney of mice after treatment with saline and nanoparticles NPs1 and NPs2. i) Histopathological H&E staining of liver, spleen, lung, and kidney tissues of healthy mice and infected mice treated with saline and peptide nanoparticles NPs1 and NPs2. Scale bar: 100 µm. j–l) Serum levels of IL‐6, IL‐1*β*, and TNF‐*α* in healthy mice and *E. coli‐*infected mice treated with saline and peptide nanoparticles NPs1 and NPs2. e–h, j–l) Values denote the mean ± SD, *n* = 6. The differences between the groups were determined by one‐way ANOVA followed by Tukey's post hoc analysis. Different values in the superscript indicate a significant difference (*p* < 0.05).

To determine the therapeutic effects of the self‐assembling peptide nanoparticles NPs1 and NPs2 in vivo, we used *E. coli* to establish systemic sepsis model. Sepsis is a systemic inflammatory response syndrome caused by bacteria invading the body; it is a common systemic infection with a high fatality rate.^[^ ^39^ ^]^ One of the pathogeneses of sepsis is that endotoxins released by gram‐negative bacteria cause an uncontrolled inflammatory response and immune dysfunction.^[^ ^40^ ^]^ Compared with traditional antibiotics, one of the advantages of peptide‐based antibacterial drugs in the treatment of sepsis is that the positively charged peptide nanoparticles can neutralize the negatively charged endotoxin, thereby reducing the body's inflammatory response. Furthermore, the membrane destruction mechanism of peptide nanoparticles makes it difficult for bacteria to develop drug resistance. For this reason, we first evaluated the ability of the challenge strain to develop resistance to the peptide nanoparticles NPs1 and NPs2, and we used colistin as a control. As shown in **Figure** **6**b, no spontaneous antimicrobial resistance to the peptide nanoparticles was observed during continuous passage at sub‐MIC concentrations. The results of the action mechanism investigation showed that peptide nanoparticles primarily exert antibacterial effects by penetrating and destroying bacterial membranes. Bacteria must change their membrane structure to resist attack by peptide nanoparticles. Therefore, the probability of peptide nanoparticles inducing bacterial resistance is very low. As a control, the antimicrobial activity of colistin decreased by ≈ 32–64 times after the end of the assay period. It has been reported that the drug resistance of colistin is related to the transmission of the plasmid‐mediated gene MCR1 (mobile colistin resistance) between different strains, as well as the addition of cationic groups (e.g., L‐Ara4N and pEtN) to LPS. Subsequently, to clarify the sterilization procedures of the peptide nanoparticles, we measured their time‐kill curves against *E. coli*. As shown in Figure 6c,d, peptide nanoparticles NPs1 and NPs2 could not completely kill *E. coli* within 120 min at a concentration of 8 × 10^−6^ m. Under exposure to the peptide nanoparticles at a concentration of 16 × 10^−6^ m, almost all *E. coli* in the solution were killed after 120 min, though the sterilization speed of the peptide nanoparticles was not significantly improved.

Based on the results of the in vivo toxicity study, we selected the safe dose of peptide nanoparticles (15 mg kg^−1^) for in vivo activity evaluation (Figure 6a). After the mice were infected with *E. coli*, the treatment group was treated with 15 mg kg^−1^ peptide nanoparticles NPs1 or NPs2, and the saline treatment group was used as a control. After 12 h of infection, the mice were autopsied and the colonies in the organs were counted. Compared with the saline treatment, the bacterial loads in the liver, kidney, spleen, and lung were significantly reduced after treatment with peptide nanoparticles NPs1 and NPs2 (Figure 6e–h). Moreover, compared with the saline treatment group, the levels of pro‐inflammatory factors TNF‐*α*, interleukin‐6 (IL‐6), and interleukin‐1*β* (IL‐1*β*) in the serum of mice in the peptide nanoparticle treatment group were significantly reduced (Figure 6j–l). These cytokines are key factors affecting the inflammatory response in acute sepsis. Considering the action mechanism of peptide nanoparticles, combining them with LPS to reduce the circulation of endotoxins in vivo may limit the immunogenic potential of mice.^[^ ^41^ ^]^ These results indicate that peptide nanoparticles not only exert direct antibacterial effects but also have immunomodulatory properties. These effects complement each other and help prevent systemic bacterial infections. Hematoxylin and eosin (H&E) staining showed pathological changes in the tissues of infected mice, including hepatocyte damage, vacuoles around the glomerulus, and inflammatory cell infiltration (Figure 6i). After peptide nanoparticle treatment, tissue damage was largely prevented or restored.

The policy banning the addition of feed antibiotics in feed and breeding in animal husbandry, results in huge demand for antibiotic alternatives for young animals such as weaning piglets. In addition, as an experimental animal, the anatomy and physiology of pigs are closer to humans, and immune responses comparable to humans.^[^ ^42^ ^]^ Moreover, using pigs as a model can better reflect the efficacy of antibacterial drugs and the interaction of pathogens and hosts. Therefore, the in vivo activity of peptide nanoparticles was further verified with piglets as target animals. As shown in Figure S12 in the Supporting Information, compared with the saline treatment group, the bacterial loads in the organs of the peptide nanoparticles NPs1 and NPs2 treatment groups was significantly reduced, and the levels of inflammatory factors were alleviated. Additionally, infected piglets developed liver sinus enlargement, minor hemorrhage in the spleen, hemorrhage, and edema in the lungs. The liver, spleen, lung, and kidney of piglets treated with peptide nanoparticles were almost no different from the control group.

Collectively, these data suggest that chimeric peptide nanoparticles can reduce the tissue damage and inflammatory factor disorders induced by systemic bacterial infections, and they are beneficial for the treatment of sepsis and bacterial infections.

## 3 Conclusion

In this study, we report upon a self‐assembling chimeric peptide nanoparticle composed of alkyl chains, hydrophobic units, positively charged units, and PEG units. This research focused on identifying the antibacterial activity, stability, and biocompatibility of chimeric peptide nanoparticles in vitro, and we discussed its bactericidal mechanism in detail. The results confirmed that the self‐assembling peptide nanoparticles NPs1 and NPs2 exhibited broad‐spectrum antibacterial activity, good biocompatibility, and excellent salt and protease stability. Meanwhile, peptide nanoparticles NPs1 and NPs2 have shown application potential in the treatment of systemic infections. The mechanism research results indicate that peptide nanoparticles interact with negatively charged components on the membrane surface, destroying the entire membrane structure, and affect the cell cycle, eventually causing the bacterial content to leak and die. Altogether, these findings may provide a theoretical basis for the design of efficient and safe peptide‐based antibacterial nanomaterials, as well as promising candidates for solving the antibiotic shortage without causing bacterial resistance, in both human health and animal husbandry.

## 4 Experimental Section

### Synthesis of the Peptide Nanoparticles

The designed peptide nanoparticles were synthesized by Sangon Biotech (Shanghai, China) and purified by RP‐HPLC to a purity exceeding 95%. The fidelity and precise molecular masses of the peptides were measured by MALDI‐TOF‐MS.

### Determining the CMC of Self‐Assembling Peptide Nanoparticles

The hydrophobic fluorescent probe ANS was used to determine the CMC of the peptide nanoparticles, as previously described.^[^ ^43^ ^]^ The peptide nanoparticles were diluted in PBS (10 × 10^−3^ m) to a final concentration range of 1 × 10^−6^–256 × 10^−6^ m. Subsequently, ANS with a final concentration of 10 × 10^−6^ m was added to the peptide nanoparticles, and the product was incubated for 1 h. Then, a microplate reader was used to measure the fluorescence spectrum of the peptide solution under an excitation wavelength of 360 nm, an emission wavelength range of 420–670 nm, and an interval of 5 nm. Finally, the corrected fluorescence intensity at a wavelength of 485 nm and the logarithm of peptide concentration for the plot were used. CMC was determined as the intersection of the two fitted straight lines.

### TEM and AFM Observation of Peptide Nanoparticles

The morphologies of the peptide nanoparticles were observed using TEM.^[^ ^13^, ^44^ ^]^ The peptide solution was diluted to 128 × 10^−6^ m in PBS (10 × 10^−3^ m, pH 7.4) and then placed on a 100‐mesh copper‐plated grid; after 5 min, the excess solution was absorbed with filter paper and air‐dried for 20 min. Subsequently, the sample was negatively stained with 1% phosphotungstic acid for 30 s, the excess dye was absorbed, and the sample was air‐dried and observed using a TEM.

For AFM sample preparation, 10 µL of the same peptide sample as used for the TEM experiment was uniformly coated on the mica sheet; the sample was waited for to be completely air‐dried and was observed using AFM.

### Dynamic Light Scattering

The peptide nanoparticles were diluted with PBS (10 × 10^−3^ m, pH 7.4) to a concentration of 128 × 10^−6^ m, and the particle size distribution of the samples was measured by dynamic laser light scattering (DynaPro NanoStar).

### Determining of the Secondary Structures of Peptide Nanoparticles

The secondary structure of peptide nanoparticles was recorded by circular dichroism (CD) spectrometer.^[^ ^45^ ^]^ The spectra of the peptide nanoparticles (final concentration: 50 × 10^−6^ m) were measured in 10 × 10^−3^ m PBS and 30 × 10^−3^ m SDS. The data were converted to molar ellipticity ([*θ*], deg cm^2^ dmol^−1^), which is expressed as follows: observed ellipticity/peptide nanoparticle concentration (mM) × path length (mm).

### Antimicrobial Activity Assay

The MICs of the self‐assembling peptide nanoparticles were determined using the modified broth microdilution method.^[^ ^46^ ^]^ The bacteria were resuspended in a logarithmic growth phase in a Mueller–Hinton broth (MHB) medium, the optical density (OD) at a wavelength of 600 nm was adjusted to ≈ 0.4, and the suspension was diluted 1000 times with MHB. In a 96‐well plate, the peptides were diluted via multiple dilutions using a 0.2% bovine serum albumin solution to a concentration range of 0.25 × 10^−6^–128 × 10^−6^ m. Subsequently, the diluted bacterial solution and peptide solution were mixed in a 1:1 ratio in a 96‐well plate and incubated at 37 °C. After 18–24 h, the absorbance at 492 nm was measured using a microplate reader. The MIC value was defined as the lowest peptide nanoparticle concentration without bacterial growth. Each test was performed in duplicate and repeated three times.

### Cytotoxicity Assay

3‐(4,5‐dimethylthiazol‐2‐yl)‐2,5‐diphenyltetrazolium bromide (MTT) dye was used to determine the cytotoxicity of the peptide nanoparticles.^[^ ^47^ ^]^ Human embryonic kidney (HEK) 293T cells were seeded into 96‐well plates at a concentration of (1–2) × 10^5^ cells per well in high glucose DMEM and placed in a cell incubator (5% CO_2_, 37 °C) for 24 h. After discarding the medium, peptides (100 µL) were added with a concentration range of 0.125 × 10^−6^–64 × 10^−6^ m to wells 1–10 of a 96‐well plate and incubated at 37 ℃ for 2 h. Samples without peptides were used as positive controls and samples without cells were used as negative controls. Subsequently, 20 µL of MTT (5 mg mL^−1^) was added to each well before another 4 h of incubation. After discarding the supernatant, 150 µL of dimethyl sulfoxide was added to each well of the 96‐well plate, to fully dissolve the crystals; then, the absorbance at a wavelength of 570 nm was measured using a microplate reader. Each test was performed in duplicate and repeated three times. Cell viability was expressed as a percentage. For laser confocal microscope samples, after the peptides (the final concentration is 64 × 10^−6^ M) were added to a 96‐well plate, the final concentration of 5 × 10^−6^ M SYTO9 and 20 µg mL^−1^ PI dye was added to a 96‐well plate, and incubated at 37 ℃ for 2 h. Cells were imaged using a laser scanning confocal microscope. A sample without peptide nanoparticles was used as a control.

### Hemolytic Activity Assays

Fresh porcine red blood cells were used to study the hemolysis of self‐assembled peptide nanoparticles.^[^ ^48^ ^]^ The red blood cells were collected by washing with PBS (10 × 10^−3^ m, pH 7.4) until the supernatant became clear. A 10% v/v red blood cell suspension was prepared with PBS, and peptide nanoparticles with a final concentration of 128 × 10^−6^ m were added to the suspension (1 mL). After gentle incubation at 37 °C for 2 h, the cells were centrifuged at 6000 rpm for 5 min, and the absorbance of the supernatant was measured at 570 nm. Two red blood cell suspensions (one containing 0.2% Triton X‐100, the other without peptide nanoparticles) were used as positive and negative controls, respectively. The hemolysis of each sample was calculated, and images were collected.

### Salt Tolerance Assay

According to a previous method, the change in the antibacterial abilities of peptide nanoparticles in the presence of salt was measured.^[^ ^49^ ^]^ The MICs of peptide nanoparticles against *E. coli* ATCC25922 and *S. aureus* ATCC6538 were measured in the presence of physiological salts (150 × 10^−3^ m NaCl, 4.5 × 10^−3^ m KCl, 6 × 10^−6^ m NH_4_Cl, 8 × 10^−6^ m ZnCl_2_, 1 × 10^−3^ m MgCl_2_, and 4 × 10^−6^ m FeCl_3_). Each test was performed in duplicate and repeated three times.

### Protease Stability Assay

An equal volume of peptide nanoparticles (2560 × 10^−6^ m) and pepsin, trypsin, or chymotrypsin (8 mg mL^−1^) was mixed and incubated at 37 °C for 1 h. Then, the MICs of peptide nanoparticles against *E. coli* ATCC25922 and *S. aureus* ATCC6538 were measured using the above‐mentioned method. Each test was performed in duplicate and repeated three times.

For RP‐HPLC, an equal volume of peptide nanoparticles (2560 × 10^−6^ m) and pepsin, trypsin, or chymotrypsin (4 or 8 mg mL^−1^) was mixed. The mixed solution was incubated at 37 °C for a predetermined time (0, 2, 4, or 8 h), and the protease was completely inactivated by heating in boiling water at 100 °C for 5 min. The concentration of peptide nanoparticles in the samples was then diluted to 256 × 10^−6^ m, and the samples were analyzed using an RP‐HPLC system equipped with an Acchorm Unitary C18 column (5 µm, 100 Å, 4.6 mm ×  250 mm). A gradient of 42% to 96% acetonitrile in water with 0.1% trifluoroacetic acid was used as the mobile phase at a flow rate of 0.8 mL min^−1^. UV detection was performed at a wavelength of 214 nm.

### LPS and LTA Binding Assay

The fluorescent probe BODIPY‐TR cadaverine (BC) was used to determine the abilities of the peptide nanoparticles to bind to the LPS of *E. coli* and the LTA of *S. aureus*.^[^ ^50^ ^]^ The BC (5 µg mL^−1^) probe and LPS (50 µg mL^−1^) or LTA (50 µg mL^−1^) were mixed in Tris buffer (50 × 10^−3^ m, pH 7.4) and incubated in the dark for 4 h at 37 °C. The peptide solution was serially diluted in a 96‐well plate to produce a concentration in the range of 8 × 10^−6^–128 × 10^−6^ m. Then, LPS or LTA probes were mixed with an equal volume of peptide solution and incubated for 1 h. The fluorescence intensity at an excitation wavelength of 580 nm and emission wavelength of 620 nm was recorded using a microplate reader. Each test was performed in duplicate and repeated three times.

### Surface Plasmon Resonance (SPR) Assay

The real‐time interaction between the peptide and LPS (or LTA) was determined using a highly sensitive biomolecular interaction analysis system (Biacore S200).^[^ ^51^ ^]^ The peptides NPs1 and NPs2 were covalently immobilized on Channels 2 and 4 of the CM5 chip, respectively, with Channels 1 and 3 as reference channels. The peptide NPs1 (or NPs2) was immobilized at ≈ 2000 resonance units using an amine coupling kit, and the unreacted surface was blocked with ethanolamine. LPS (or LTA) in the buffer (10 × 10^−3^ m phosphate buffer, pH 7.4) was flowed over the chip surface at a concentration of 1.56–25 µg mL^−1^ and a flow rate of 10 µL min^−1^. After each sample was measured, the chip was regenerated with regeneration reagents (50 × 10^−3^ m NaOH containing 0.05% w/v SDS). Each data point denotes the result of subtracting the signal from the reference channel.

### Outer Membrane Integrity Assay

The outer membrane permeability of *E. coli* ATCC25922 induced by peptide nanoparticles was measured using the fluorescent probe *N*‐phenyl‐1‐naphthylamine (NPN).^[^ ^52^ ^]^ *E. coli* ATCC25922 in the logarithmic growth stage was diluted with 5 × 10^−3^ m HEPES (4‐(2‐hydroxyethyl)‐1‐piperazineethanesulfonic acid) buffer until the OD was 0.2 at a wavelength of 600 nm. Subsequently, NPN at a final concentration of 10 × 10^−6^ m was added to the bacterial suspension. Next, 100 µL of the bacterial suspension was mixed with 100 µL of peptide nanoparticles (final concentration range: 2 × 10^−6^–64 × 10^−6^ m) in a 96‐well plate. The fluorescence values were measured at an excitation wavelength of 350 nm and emission wavelength of 420 nm using a microplate reader. The 20 µg mL^−1^ polymyxin B treatment group was used as a positive control, and the initial fluorescence of NPN in the absence of peptide was used as a negative control. Each test was performed in duplicate and repeated three times.

### Cytoplasmic Membrane Depolarization Assay

The fluorescent probe DiSC_3_‐5 was used to determine the changes induced by peptide nanoparticles in the cytoplasmic membrane potential of *E. coli* ATCC25922 and *S. aureus* ATCC6538.^[^ ^53^ ^]^ The bacterial cells were resuspended in the logarithmic growth phase in 5 × 10^−3^ m HEPES buffer (pH 7.4, containing 40 × 10^−3^ m glucose and 200 × 10^−3^ m K^+^), and the OD was adjusted at a wavelength of 600 nm to 0.1. Subsequently, 0.8 × 10^−6^ m DiSC_3_‐5 was added to the bacterial suspension and incubated in the dark for 90 min. Next, the peptide solution was serially diluted in a 96‐well plate to produce a concentration in the range of 16 × 10^−6^–32 × 10^−6^ m. After mixing the peptide solution with the bacterial suspension (1:1, v/v), a microplate reader was used to detect the fluorescence changes at an excitation wavelength of 620 nm and an emission wavelength of 670 nm.

### Laser Scanning Confocal Microscopy Imaging

To further determine the integrity of the *E. coli* ATCC25922 and *S. aureus* ATCC6538 membranes, the bacteria were stained with SYTO9/PI dye and observed under a laser confocal microscope.^[^ ^54^ ^]^ The bacteria in the logarithmic growth phase were centrifuged and resuspended in PBS (10 × 10^−3^ m, pH 7.4) to an OD of 0.1 at a wavelength of 600 nm. The bacterial suspension and peptide nanoparticles (final concentration: 16 × 10^−6^ m) were mixed 1:1 in a 96‐well plate and added to a final concentration of 5 × 10^−6^ m SYTO9 and 20 µg mL^−1^ PI dye, before being incubated at 37 °C for 2 h. Bacterial cells were imaged using a laser scanning confocal microscope. A sample without peptide nanoparticles was used as a control.

### Flow Cytometry Analysis

*E. coli* ATCC25922 and *S. aureus* ATCC6538 in the logarithmic growth stage were diluted in PBS (10 × 10^−3^ m), and the OD was adjusted at a wavelength of 600 nm to 0.1. The peptide nanoparticles were serially diluted in a 96‐well plate to produce a concentration in the range of 8 × 10^−6^–16 × 10^−6^ m. Subsequently, the diluted bacterial solution (100 µL) and peptide nanoparticles (100 µL) were mixed in a 96‐well plate and incubated at 37 °C for 1 h. PI was added at a final concentration of 20 µg mL^−1^ and incubated at 37 °C for 30 min. The PI dye (P8080) was purchased from Beijing Solarbio Science & Technology Co., Ltd. Finally, the fluorescence was collected by flow cytometry. A sample without peptide nanoparticles was used as a control.

Bacterial suspensions prepared in the same way were used for the cell cycle analysis.^[^ ^55^ ^]^ Peptide nanoparticles (NPs1 and NPs2) at a final concentration of 8 × 10^−6^ m were added to the bacterial suspension and incubated at 37 °C for 1 h. After washing with PBS, the cells were centrifuged (8000 rpm, 5 min) and fixed with 70% ethanol overnight. To eliminate the effects of RNA, the cells were treated with 200 µg mL^−1^ RNase A and incubated at 37 °C for 1 h. Cells were washed and resuspended in PI (20 µg mL^−1^) solution and incubated in the dark for 30 min. The cell cycle was then measured using flow cytometry. Each test was repeated three times.

### SEM and TEM Observation of the Morphological Changes of Bacteria

*E. coli* ATCC25922 and *S. aureus* ATCC6538 in the logarithmic growth phase were resuspended in PBS (10 × 10^−3^ m, pH 7.4), and the OD at a wavelength of 600 nm was adjusted to 0.2; then, the resulting product was treated with peptide nanoparticles in a concentration of 16 × 10^−6^ m for 2 h at 37 °C. Subsequently, the sample was centrifuged at 12 000 rpm, the supernatant was discarded, and 2.5% glutaraldehyde was added; the sample was then fixed overnight at 4 °C. For SEM observations, the samples were continuously processed with 50%, 70%, 90%, and 100% ethanol, as well as 50% and 100% *tert*‐butanol. Subsequently, the sample was dried using liquid CO_2_, sputtered with gold‐palladium, and observed using SEM. For TEM observations, the samples were post‐fixed with 2% osmium tetroxide for 70 min, followed by continuous treatment with 50%, 70%, 90%, and 100% ethanol, as well as 50% and 100% acetone. Subsequently, the sample was coated with epoxy resin, sectioned with an ultramicrotome, stained with uranyl acetate and lead citrate, before being observed using TEM.

### Source of Animal and Ethics Statement

All 4 weeks old SPF female mice were provided by Beijing HFK Bioscience Co., Ltd. Weaned female piglets (Dorec × Landrace × Large white) were provided by Chongqing Hechuan Dekang Pig Breeding Co., Ltd. All animals were raised and handled in compliance with the Chinese laws and guidelines (Protocol GKFCZ2001545), EU Directive 2010/63/EU for animal experiments, and the China Agricultural University regulations concerning the protection of animals used for scientific purposes (2010‐SYXK‐0037). All experimental animal procedures were approved by the Animal Care and Use Committee of the China Agricultural University.

### In Vivo Toxicity Assessment

Healthy female C57BL/6 mice (weight is about 20 g) were randomly divided into five groups with six replicates per group. Mice were acclimated for 3 d in an environment of suitable temperature (20–24 °C), humidity (50–60%), and alternating light and dark. During the assay, the first group of mice was intraperitoneally injected with 150 µL of saline (once daily), and the second to fifth groups of mice were intraperitoneally injected with 150 µL nanoparticles NPs1 or NPs2 at doses of 15 and 30 mg kg^−1^ (once daily). Continuous injection was given for 7 days. At 12 h after the last administration, the mice were euthanized using mild ether anesthesia. Blood samples were taken from the mice's orbital vein, and serum was analyzed using an automatic biochemical analyzer. The kidneys and livers of the mice were taken, weighed, and fixed with 4% paraformaldehyde; then, histological analysis was performed via H&E staining.

### Drug Resistance Assays

The development of drug resistance in peptide nanoparticles was also evaluated.^[^ ^56^ ^]^ For comparison, the development of resistance to an antibiotic (colistin) was determined. As mentioned above, the MICs of NPs1, NPs2, and colistin against *E. coli* ATCC25922 were determined. After incubating the 96‐well plate for 24 h, the bacterial solution was taken in the sub‐MIC well and diluted 1000 times with fresh MHB medium before performing the next round of MIC assays. This procedure was repeated for 25 d. Each test was repeated three times.

### Time‐Kill Curve Assay

Logarithmic phase *E. coli* ATCC25922 cells were diluted to ≈ (2–3) × 10^5^ CFU mL^−1^ and incubated with peptide nanoparticles NPs1 or NPs2 at a concentration of 8 × 10^−6^ or 16 × 10^−6^ m in PBS (10 × 10^−3^ m, ph=7.4). At different time intervals (0, 3, 5, 15, 30, 60, and 120 min), the bacterial suspension was diluted to an appropriate multiple and plated on Mueller–Hinton agar (MHA) plates. After the plates were incubated for 24 h in an incubator at 37 °C, the bacterial colonies were counted. Each test was repeated three times.

### In Vivo Activity Assessment

Healthy female C57BL/6 mice (weighing ≈ 20 g) were randomly divided into four groups with eight replicates per group. *E. coli* ATCC25922 was diluted with saline to produce an absorbance of 0.2 at a wavelength of 600 nm; then, 100 µL of bacterial suspension was intraperitoneally injected into the first three groups of healthy mice to establish a sepsis model. The remaining group served as the untreated control group. One hour after the mice were infected, the first three groups of mice were injected intraperitoneally with 150 µL of saline, NPs1 (15 mg kg^−1^), or NPs2 (15 mg kg^−1^). After 12 h, all mice were euthanized using ether inhalation narcosis. Blood was collected from the orbital vein of the mice, and using an enzyme‐linked immunoassay to analyze cytokine levels in the serum. The inflammatory factor kit was purchased from Shanghai Hengyuan Biological Technology Co., Ltd. Additionally, the liver, kidney, spleen, and lung of each mouse were collected for weighing. Two of each group were used for histological examination, and the remainder were used for a quantitative bacterial study.

Healthy female Dorec × Landrace × Large white weaned piglets (weight ≈ 8 kg) were randomly divided into four groups of five pigs per group. 8 mL of *E. coli* ATCC25922 (OD_600_ = 0.2) suspension was injected into the abdominal cavity of three groups of healthy piglets to establish a sepsis model. The remaining group served as an untreated control group. One hour after the piglet were infected, the three groups of infected piglets were injected intraperitoneally with 10 mL of saline, NPs1 (3 mg kg^−1^), or NPs2 (3 mg kg^−1^). After 12 h, all piglets were euthanized by a potassium chloride injection. Blood was collected and enzyme‐linked immunoassay was used to analyze the cytokine levels in the serum. Partial samples were obtained from the spleen, right kidney, right lung, and right liver lobe of each pig by standardized localization for weighing and viable counts. The remaining organ samples from two pigs in each group were used for histological examination.

### Statistical Analysis

Data are presented as mean ± standard deviation (SD). Statistical analysis was performed using one‐way analysis of variance (ANOVA) followed by Tukey's post hoc analysis. The Graphpad prism and Origin software was used for graph plotting. Statistical significance was set at *p* < 0.05.

## Conflict of Interest

The authors declare no conflict of interest.

## Supporting information

Supporting Information

## Data Availability

Research data are not shared.
