# Supplementary material for: Designing Self‐Assembling Chimeric Peptide Nanoparticles with High Stability for Combating Piglet Bacterial Infections
Source: Adv Sci (Weinh). 2022 Mar 13;9(14):2105955. doi: 10.1002/advs.202105955 (PMC9109057; doi:10.1002/advs.202105955)
Supplement: Supplementary file 1 — Supporting Information [file ADVS-9-2105955-s001.pdf]

Designing self-assembling chimeric peptide nanoparticles with high stability for combating piglet bacterial infections

Peng Tan, Qi Tang, Shenrui Xu, Yucheng Zhang, Huiyang Fu, and Xi Ma *

State Key Laboratory of Animal Nutrition, College of Animal Science and Technology, China Agricultural University, Beijing, 100193, China.

*** Corresponding author: maxi@cau.edu.cn (XM).**

Bacterial Strains: The strains *E .coli* ATCC25922，*S .aureus* ATCC6538，*S .epidermidis* 49134，*P. aeruginosa* 27853 were obtained from Shanghai Luwei Technology co. ltd. The strains *E .coli* K88，*E .coli* K99, *C. rodentium* DBS100, and *S .aureus* CVCC1882 were obtained from China Veterinary Culture Collection Center.

Materials: Mueller−Hinton Broth (MHB) powder and Mueller-Hinton Agar (MHA) were obtained from AoBoX (China). Sodium dodecyl sulfate (SDS) was purchased from Sigma-Aldrich (China). Bovine serum albumin (BSA), pepsin, trypsin, chymotrypsin, *N*-phenyl-1-naphthylamine (NPN), 3,3-dipropylthiadicarbocyanine (DiSC$_3$-5), Triton X-100, LPS from *E. coli* 0111: B4, LTA from *S. aureus*, 4-(2-hydroxyethyl)piperazine-1-ethanesulfonic acid (HEPES) were purchased from Sigma-Aldrich (China). Propidium iodide (PI) were purchased from Beijing Solarbio Science & Technology Co., Ltd. BODIPY-TR-cadaverine (BC) and SYTO9 were obtained from Invitrogen. Methylthiazolyldiphenyl-tetrazolium bromide (MTT) was purchased form MedChemExpress. Mouse and porcine TNF-α, IL-4, IL-6, and IL-1β enzyme-linked immunosorbent assay (ELISA) Kit obtained from purchased from Shanghai Hengyuan Biological Technology Co. Ltd.

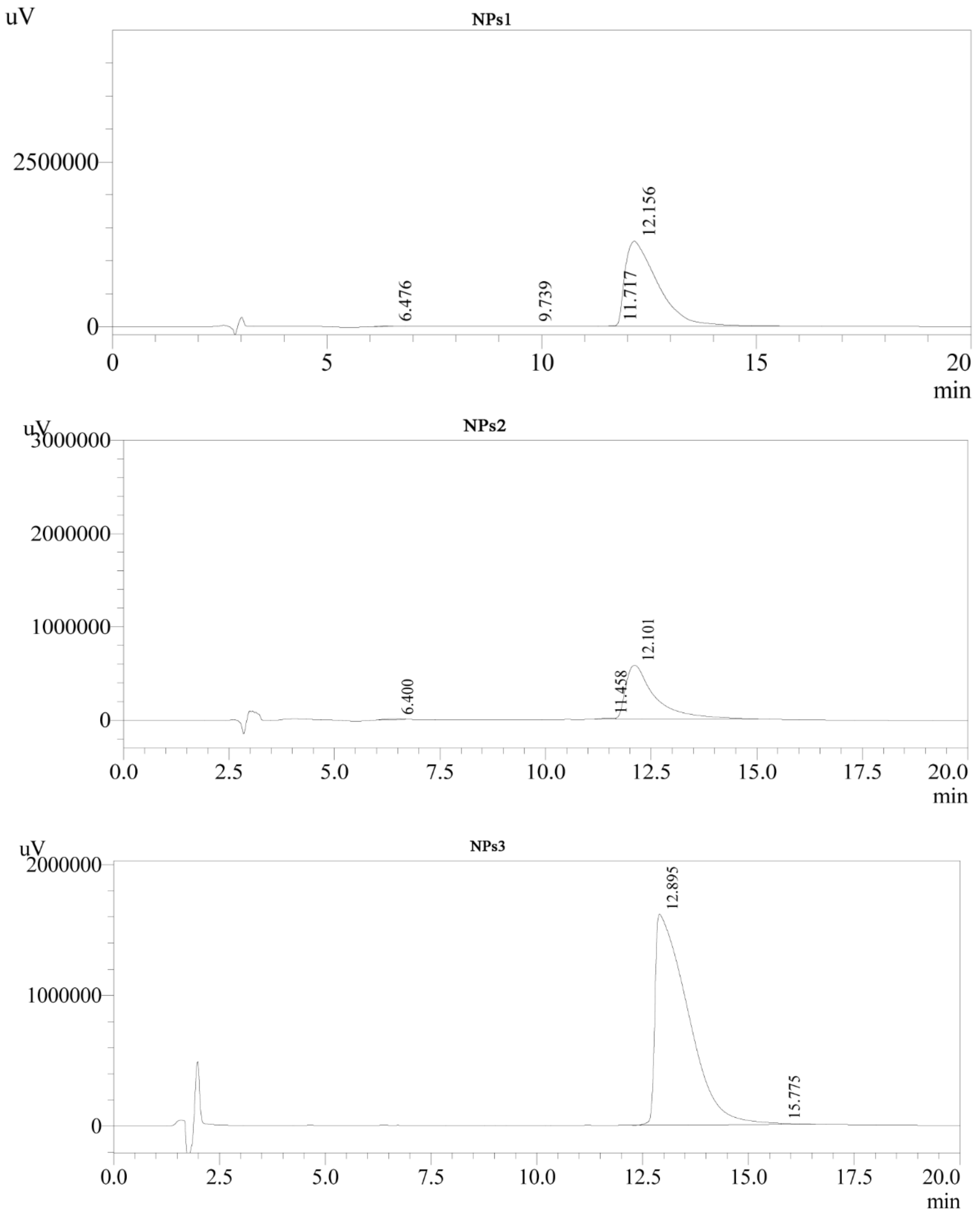

Figure S1. Reversed-phase high-performance liquid-chromatography of synthetic peptides.

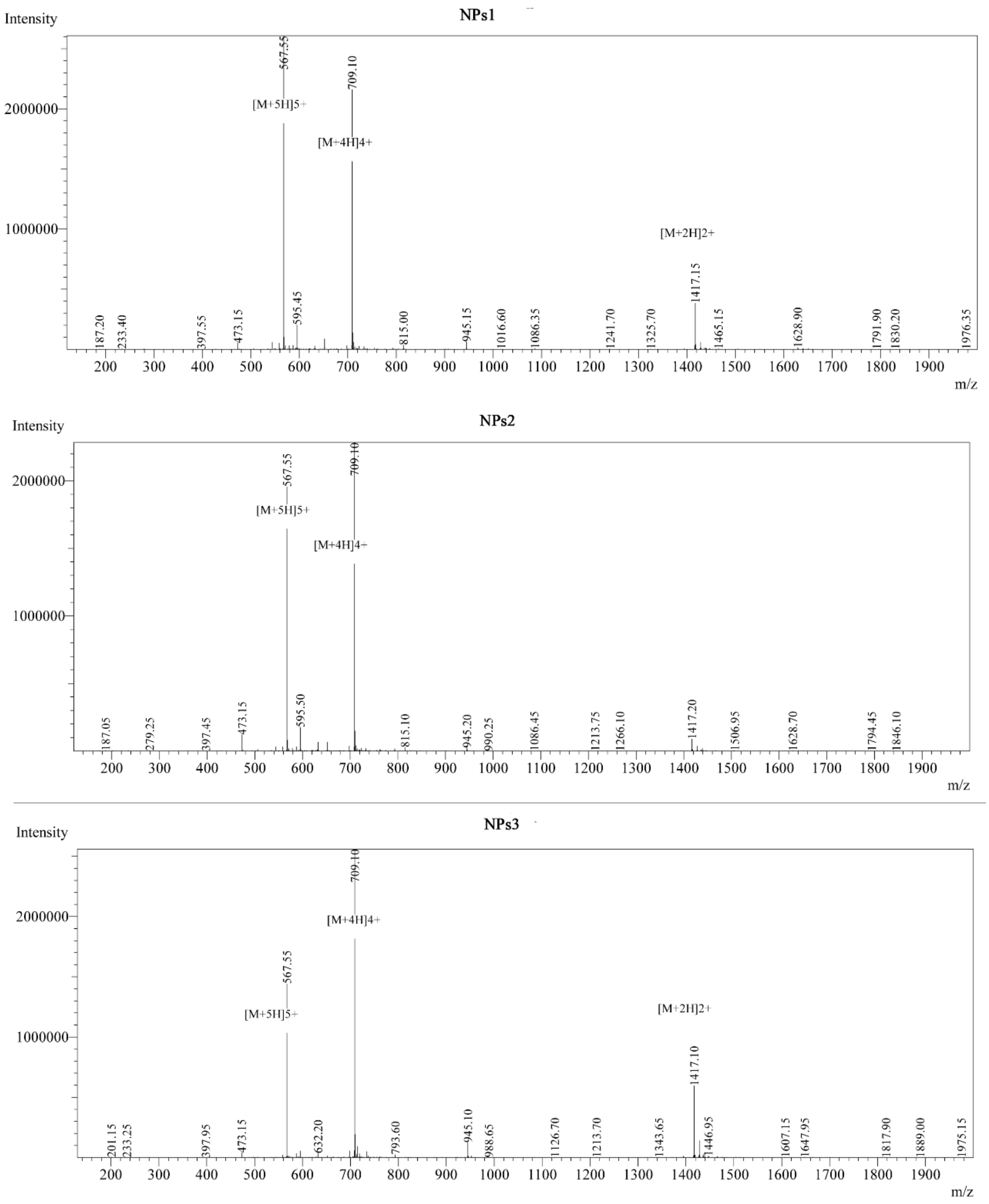

Figure S2. MALDI-TOF MS of the synthetic peptides.

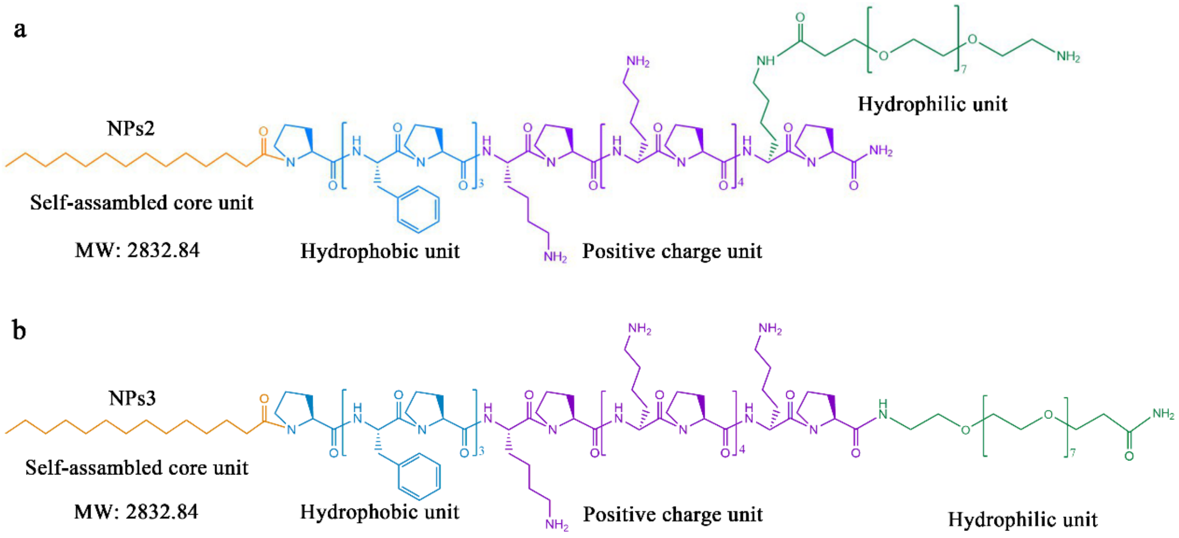

Figure S3. The molecular structure of NPs2 (a) and NPs3 (b).

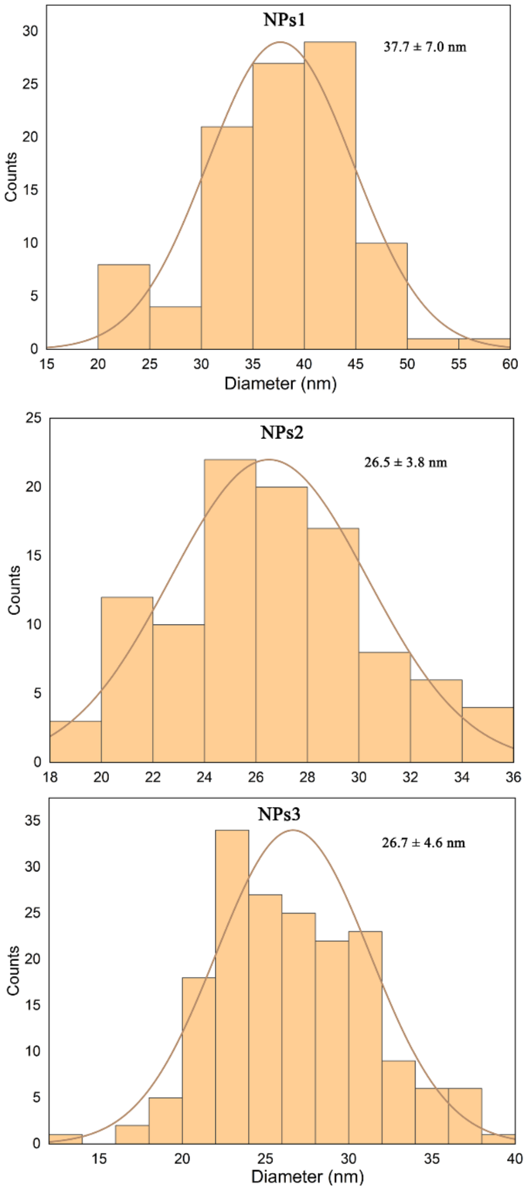

Figure S4. Diameter distributions of NPs1, NPs2, and NPs3 by TEM.

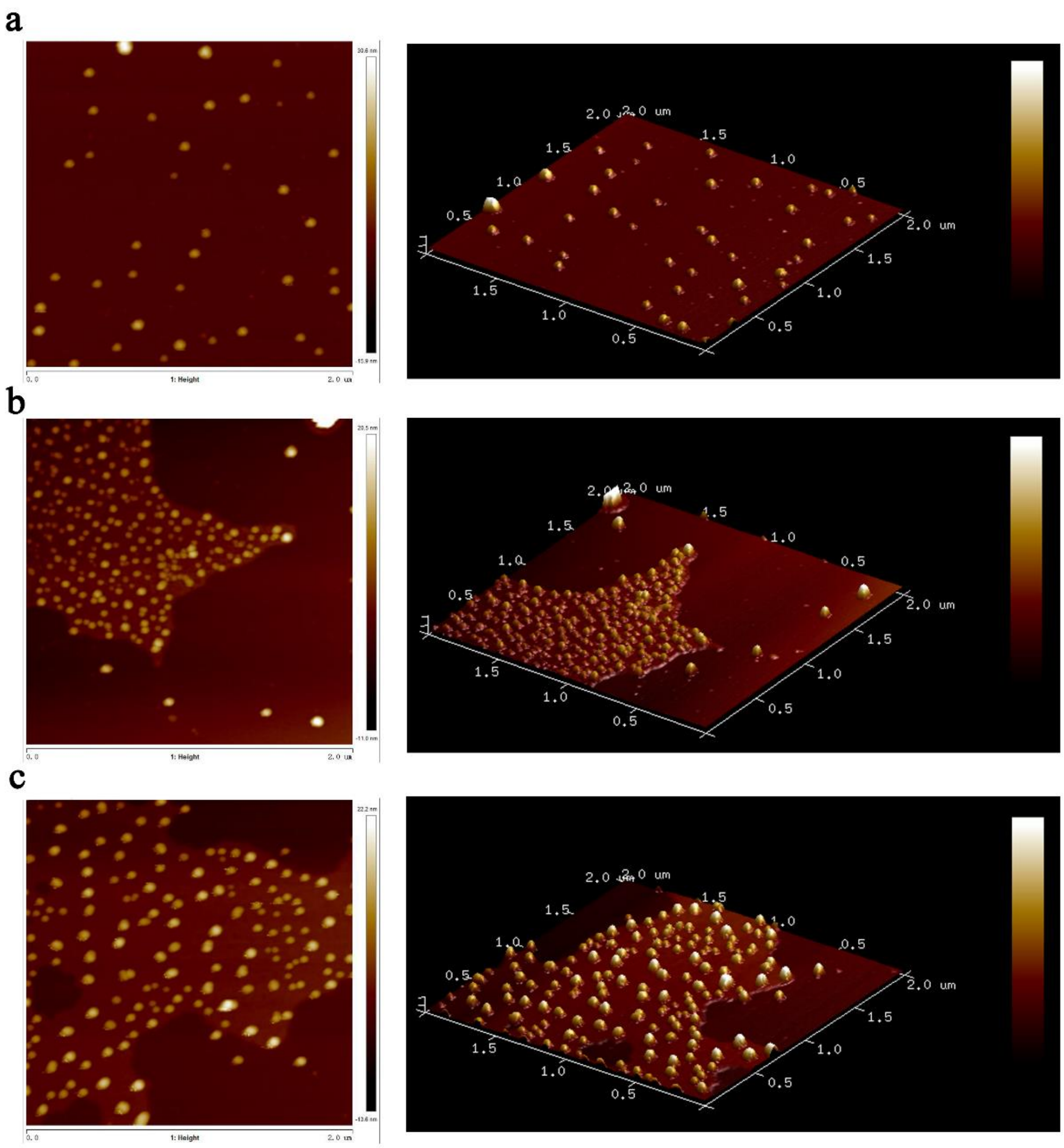

Figure S5. Atomic force microscope (AFM) images of peptide nanoparticles (a) NP1, (b) NPs2, and (c) NPs3 at a concentration of 128 μM. Scale bar: 2 μm.

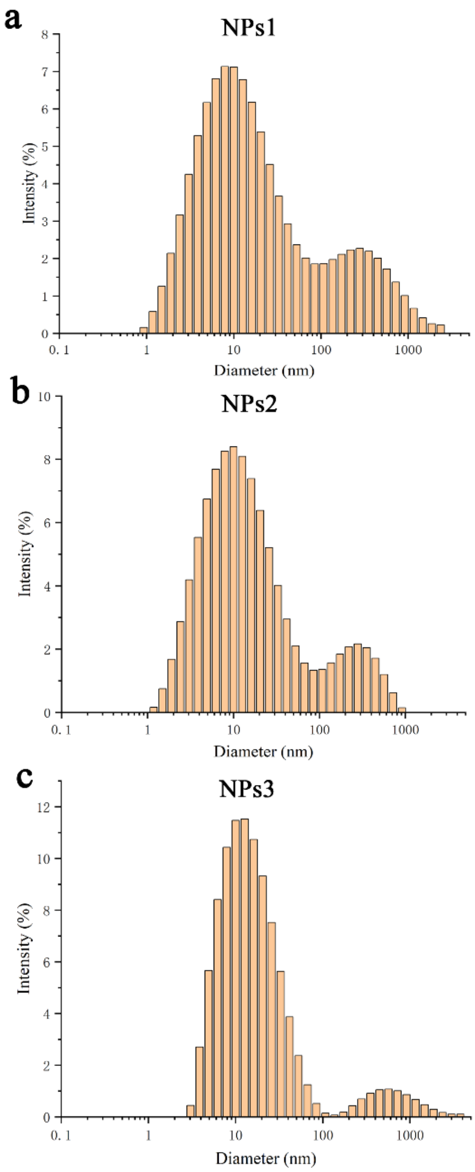

Figure S6. Dynamic light scattering (DLS) measurement of peptide nanoparticles NPs1, NPs2, and NPs3 at a concentration of 128 μM.

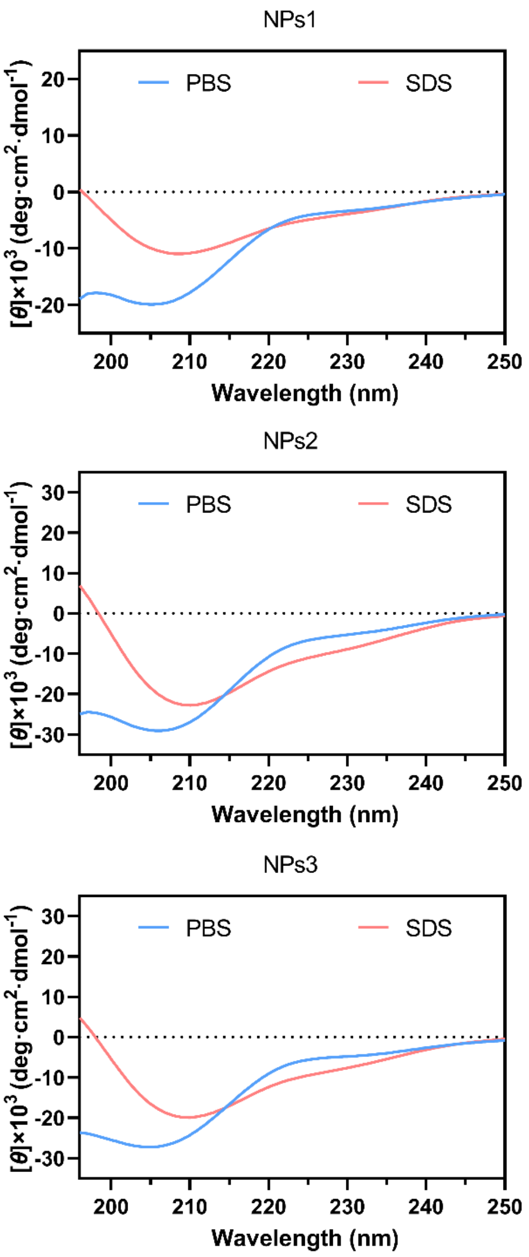

Figure S7. Circular dichroism spectrum of peptide nanoparticle NPs1, NPs2, and NPs3 in 10 mM PBS (pH 7.4) and 30 mM SDS.

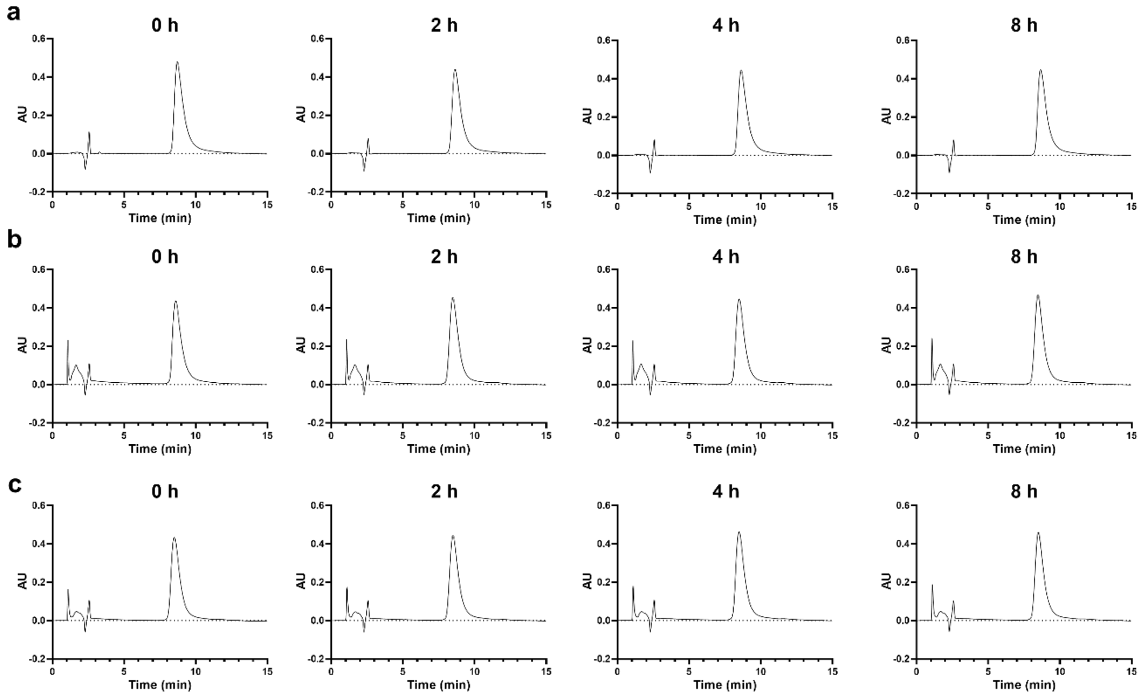

Figure S8. RP-HPLC of peptide nanoparticles NPs1 after incubation with 4 mg/mL (a) pepsin, (b) trypsin, or (c) chymotrypsin for 0, 2, 4 or 8 h.

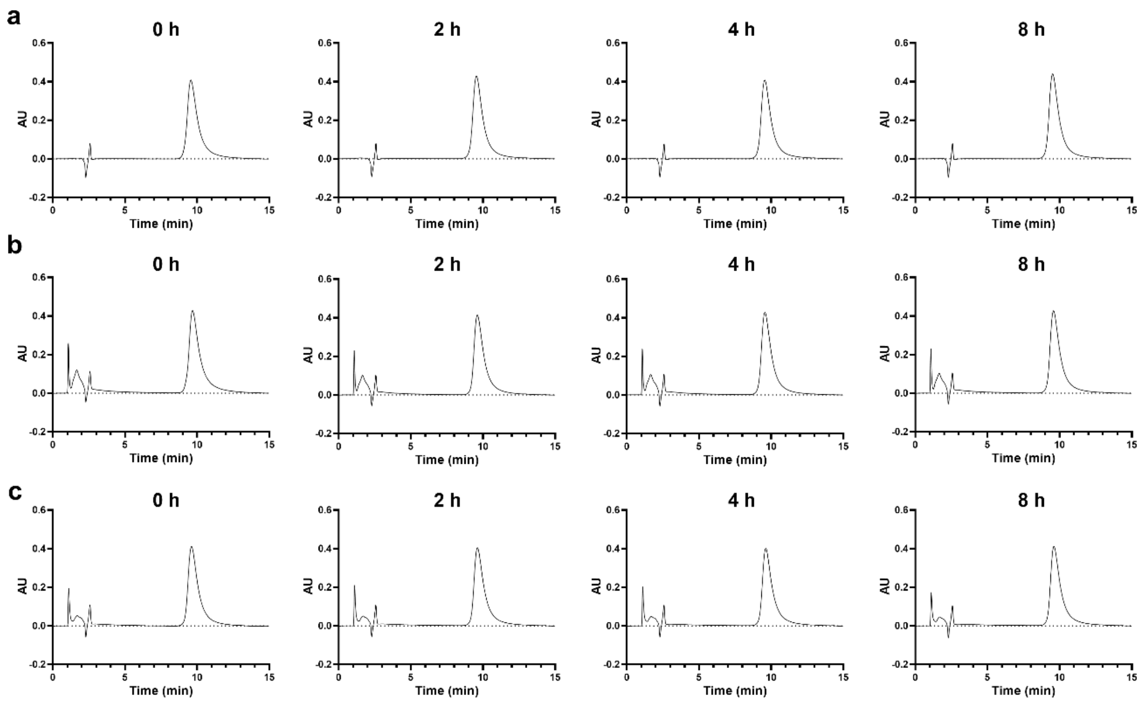

Figure S9. RP-HPLC of peptide nanoparticles NPs2 after incubation with 4 mg/mL (a) pepsin, (b) trypsin, or (c) chymotrypsin for 0, 2, 4 or 8 h.

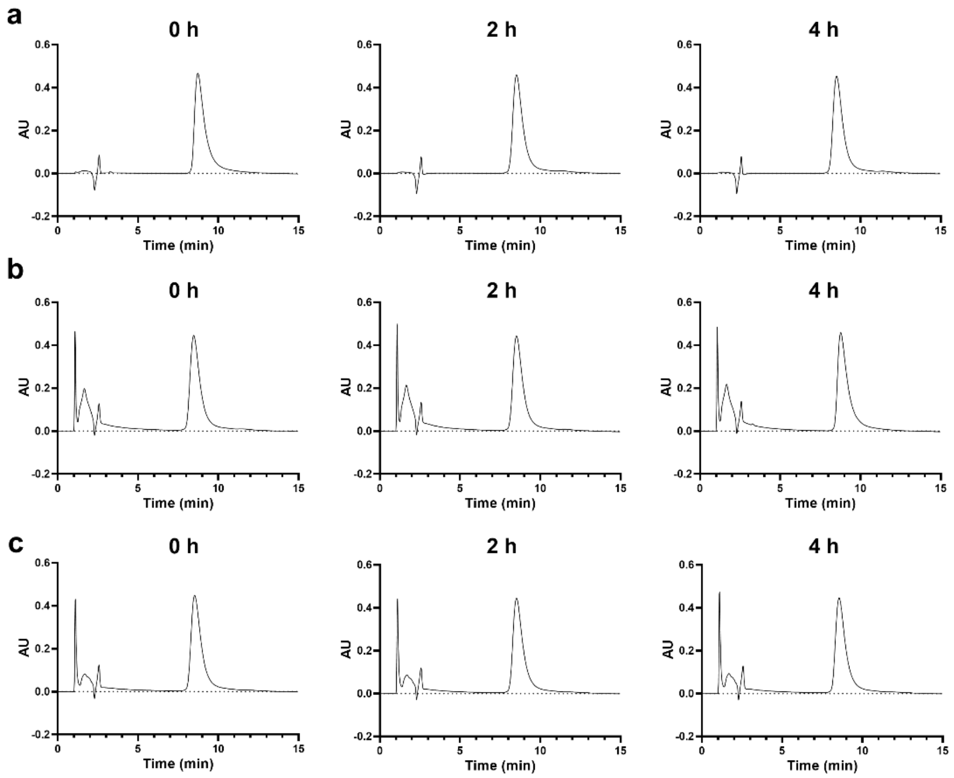

Figure S10. RP-HPLC of peptide nanoparticles NPs1 after incubation with 8 mg/mL (a) pepsin, (b) trypsin, or (c) chymotrypsin for 0, 2, or 4 h.

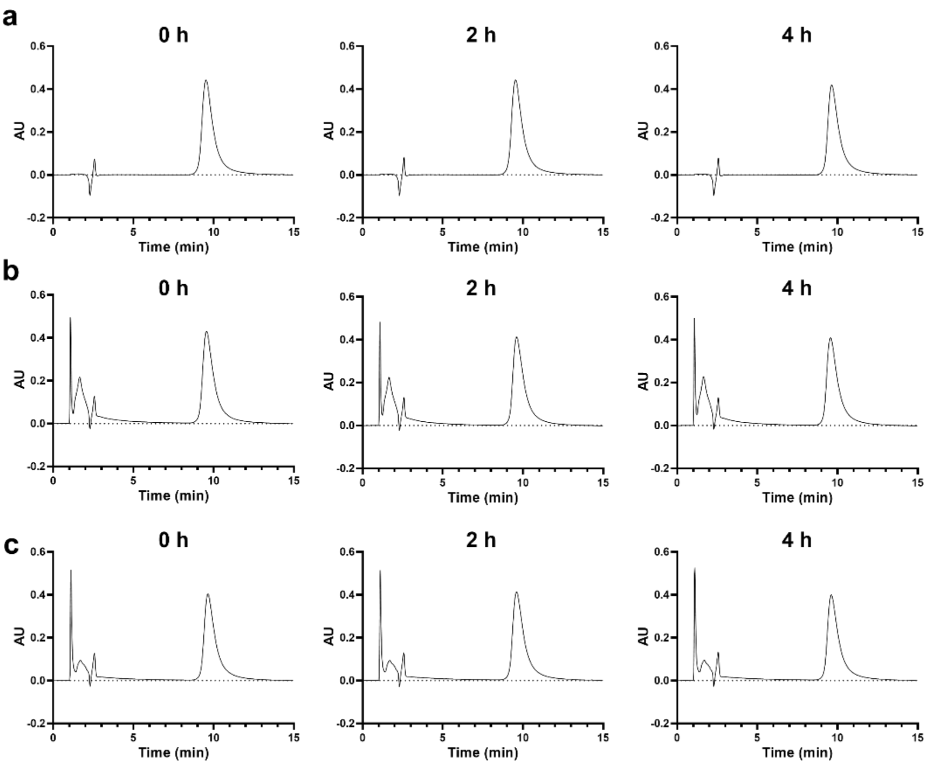

Figure S11. RP-HPLC of peptide nanoparticles NPs2 after incubation with 8 mg/mL (a) pepsin, (b) trypsin, or (c) chymotrypsin for 0, 2, or 4 h.

Table S1. Association rate constants, dissociation rate constants, and affinity constants for NPs1 and NPs2 interactions with membrane components (LPS or LTA).

Peptide	Composition	Ka (1/Ms)	Kd (1/s)	KD (M)
NPs1	LPS	750.7	0.0417	5.56×10^{-5}
NPs2	LPS	671.1	0.02066	3.08×10^{-5}
NPs1	LTA	289.3	0.007667	2.65×10^{-5}
NPs2	LTA	353.4	0.006056	1.71×10^{-5}

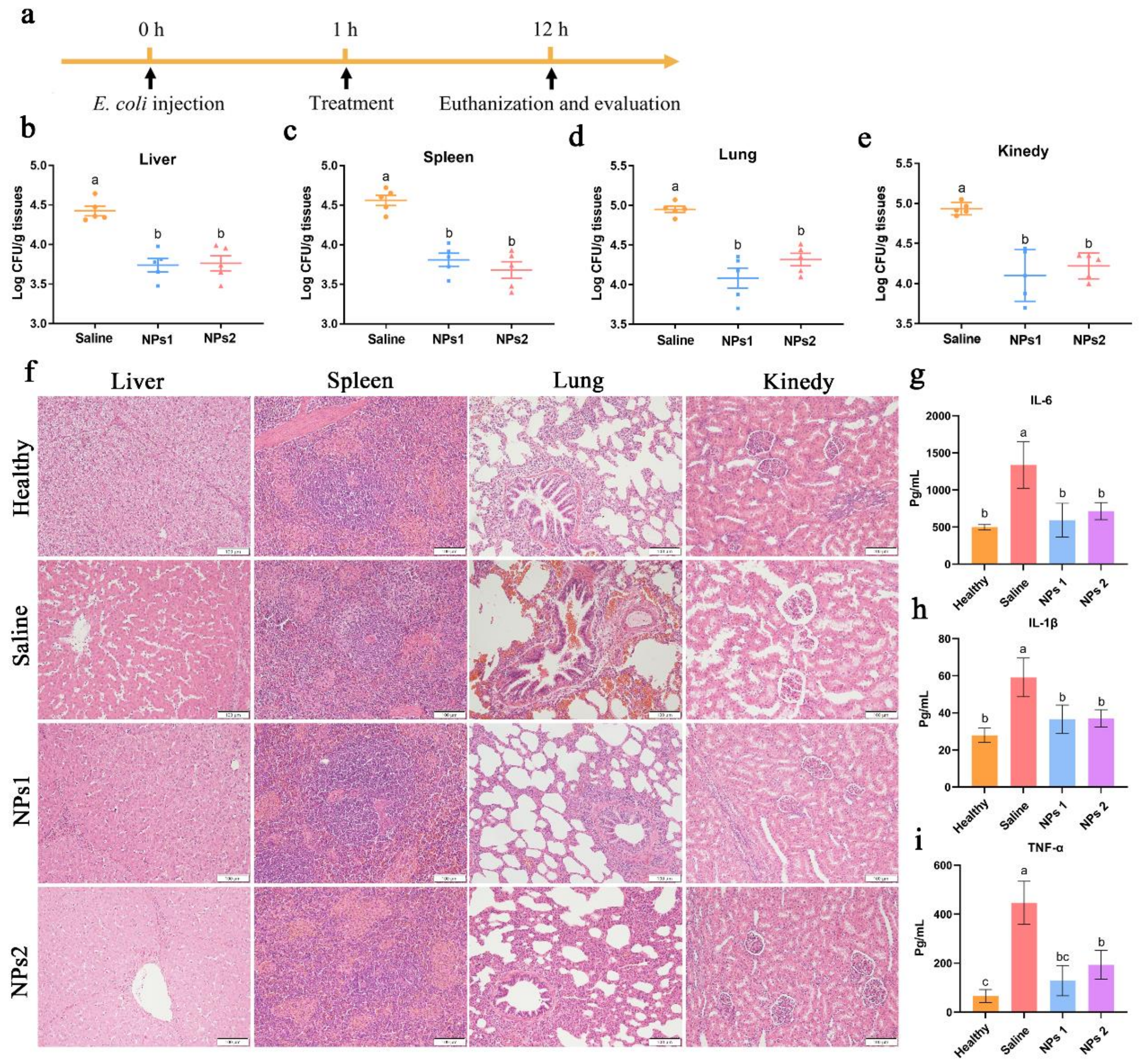

Figure S12. a) Schematic diagram of the piglet systemic infection model experiment. b-e) Bacterial loads in liver, spleen, lung, and kidney of piglets after treatment with saline and nanoparticles NPs1 and NPs2. f) Histopathological H&E staining of liver, spleen, lung and kidney tissues from healthy piglets, and infected piglets treated with saline, peptide nanoparticles NPs1 or NPs2. g-i) Serum levels of IL-6, IL-1β, and TNF-α in healthy piglets and *E. coli* infected piglets treated with saline, peptide nanoparticles NPs1, or NPs2. Values denote the mean ± SD, n = 5. The differences between the groups were determined by one-way ANOVA followed by Tukey's post hoc analysis. The different value as superscript indicates that there is significant difference ($P < 0.05$).
